# HMGB1-Promoted and TLR2/4-Dependent NK Cell Maturation and Activation Take Part in Rotavirus-Induced Murine Biliary Atresia

**DOI:** 10.1371/journal.ppat.1004011

**Published:** 2014-03-20

**Authors:** Yinrong Qiu, Jixin Yang, Wenmei Wang, Wentao Zhao, Fei Peng, Ying Xiang, Gang Chen, Tao Chen, Chengwei Chai, Shuaiyu Zheng, Daniel J. Watkins, Jiexiong Feng

**Affiliations:** 1 Department of Pediatric Surgery, Tongji Hospital, Tongji Medical College, Huazhong University of Science and Technology, Wuhan, China; 2 Institute of Organ Transplantation, Tongji Hospital, Tongji Medical College, Huazhong University of Science and Technology, Wuhan, China; 3 Department of Infectious Diseases, Tongji Hospital, Tongji Medical College, Huazhong University of Science and Technology, Wuhan, China; 4 Department of Surgery, Wayne State University, Detroit, Michigan, United States of America; Cincinnati Children's Hospital Medical Center, United States of America

## Abstract

Recent studies show that NK cells play important roles in murine biliary atresia (BA), and a temporary immunological gap exists in this disease. In this study, we found high-mobility group box-1 (HMGB1) and TLRs were overexpressed in human and rotavirus-induced murine BA. The overexpressed HMGB1 released from the nuclei of rotavirus-infected cholangiocytes, as well as macrophages, activated hepatic NK cells via HMGB1-TLRs-MAPK signaling pathways. Immature NK cells had low cytotoxicity on rotavirus-injured cholangiocytes due to low expression of TLRs, which caused persistent rotavirus infection in bile ducts. HMGB1 up-regulated the levels of TLRs of NK cells and promoted NK cell activation in an age-dependent fashion. As NK cells gained increasing activation as mice aged, they gained increasing cytotoxicity on rotavirus-infected cholangiocytes, which finally caused BA. Adult NK cells eliminated rotavirus-infected cholangiocytes shortly after infection, which prevented persistent rotavirus infection in bile ducts. Moreover, adoptive transfer of mature NK cells prior to rotavirus infection decreased the incidence of BA in newborn mice. Thus, the dysfunction of newborn NK cells may, in part, participate in the immunological gap in the development of rotavirus induced murine BA.

## Author Summary

Biliary atresia (BA) is the most common precipitating factor for liver transplantation in infants. BA is caused by the obstruction of hepatic bile ducts, leading to progressive obstructive jaundice and liver fibrosis. A well-recognized theory is that rotavirus injures biliary epithelia in a mouse model of BA, followed by attack of immunocytes, such as NK cells. We performed this research to investigate whether maturation and activation of NK cells take part in the development of BA. We identified that rotavirus induced HMGB1 release from injured bile ducts. HMGB1 induced NK cell activation in an age-dependent fashion via HMGB1-TLRs-MAPK signaling pathways. Newborn NK cells were unable to eliminate rotavirus-infected cholangiocytes, which caused persistent biliary infection; maturated NK cells were activated gradually and caused persistent biliary injury, which finally led to BA. We identify HMGB1 as an important pro-inflammatory initiator and a critical inducer for maturation of NK cells in the development of BA. HMGB1-induced activation of NK cells may, in part, plays crucial roles in the development of murine BA. Novel therapies targeting HMGB1 or TLRs in patients with BA may be applied in the future to decrease the activity of NK cells in order to inhibit the progression of BA.

## Introduction

Biliary atresia (BA), which is the most common precipitating factor leading to liver transplantation in infants [1], is a common neonatal cholangiopathy that leads to progressive hepatobiliary injury [2]. BA has been recognized as a virus-induced autoimmune disease [3], [4], in which infection by viruses, especially rotavirus, is often considered as the initiator in the pathogenesis [5]. In murine BA, rotavirus infection is followed by activation of lymphocytes and secretion of inflammatory cytokines [6], [7] targeting extrahepatic bile ducts. We have previously shown the importance of leukocyte antigen-DR [8] and osteopontin [9] in human BA. In animal studies, we have demonstrated that NF-κB regulates rhesus rotavirus (RRV)-induced BA [10], [11]. We have recently reported that rotavirus NSP4 is a crucial immunogen in BA [12]. Our previous findings have reinforced the notion that BA is a virus-induced and immune system mediated disease [4].

It is reported that high-mobility group box-1 (HMGB1) protein, which is a nuclear factor released extracellularly from immune cells or injured non-immune cells [13] and acts as an important mediator of various inflammatory responses, is demonstrated to interact with TLR2 and TLR4 [14]. However, it is poorly understood whether HMGB1 interacts with TLR2 and TLR4 to induce murine BA.

Moreover, there is a temporary immunological gap in murine BA reported by Czech-Schmidt et al [15]. In their study, when infected by RRV 12 hours after birth, the incidence of BA was 86% and a mortality of 100%. When the newborn mice were infected 24 h postpartum, 65% of newborn mice developed murine BA with a mortality of 69%; whereas no adult mice infected by RRV acquired BA [15]. In this model, various immunocytes are shown to participate in development of BA [6], [7] and some studies have demonstrated the importance of NK cells in targeting cholangiocytes after viral infection [16], [17]. However, no study has yet investigated the roles of NK cell maturation and activation in the immunological gap of murine BA.

In the present study, we found that the expression of HMGB1 is increased in human/murine BA, and the overexpressed HMGB1 is released from injured cholangiocytes and macrophages, which activates NK cells via activation of HMGB1-TLRs-MAPK signaling pathways. Immature NK cells are incapable of eliminating RRV-infected cholangiocytes in neonates, which causes persistent RRV infection in bile ducts. HMGB1 promotes maturation of NK cells as mice age, leading to an increased and persistent immune response in cholangiocytes, which induces BA. On the other hand, the activation of NK cells of adult mice is increased and their mature NK cells eliminate RRV-infected cholangiocytes shortly after infection. Therefore, in RRV-infected adult mice, the biliary tracts are not damaged and BA does not develop. Thus, immature NK cells take part in the immunological gap in the development of RRV-induced murine BA.

## Results

### Expression of HMGB1, TLR2 and TLR4 are increased in livers of patients with BA

**Figure 1:**
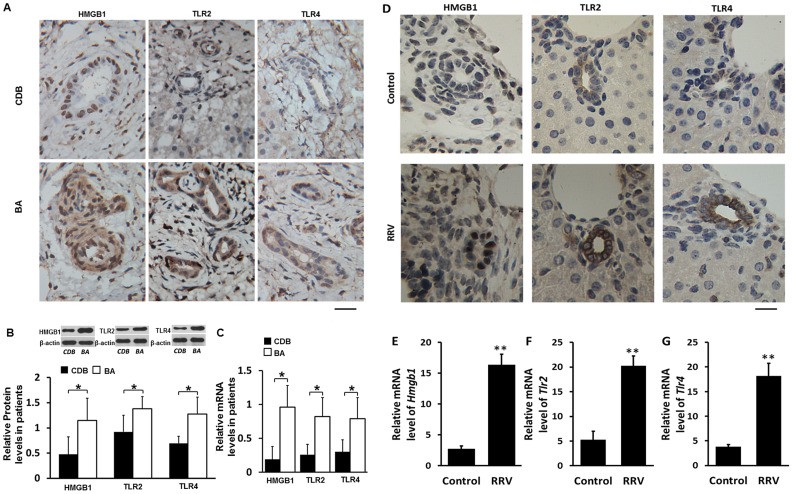
Expression of HMGB1, TLR2 and TLR4 in livers of infants with biliary atresia (BA) and in bile ducts of mice challenged with RRV. (**A**) Paraffin sections of liver tissues from infants at the time of operation for congenital dilation of the bile duct (CDB) (N = 5) or BA (N = 9) immunostained with anti-HMGB1, anti-TLR2 or anti-TLR4 antibody. Brown staining represents positive signals. The scale bar = 20 µm. (**B**) Protein levels of HMGB1, TLR2 and TLR4 detected by western blotting in liver tissues from patients with CDB or BA. The protein levels are normalized to β-actin. (**C**) mRNA levels of HMGB1, TLR2 and TLR4 detected by realtime RT-PCR. The data are normalized to *GAPDH*. (**D**) All mice were injected with 50 µl vehicle medium or 50 µl RRV supernatant intraperitoneally within 12 hours after birth and were euthanized 7 days later. Paraffin sections of livers were immunostained with anti-HMGB1, anti-TLR2 or anti-TLR4 antibody. The scale bar = 15 µm. (**E, F** and **G**) mRNA levels of HMGB1, TLR2 and TLR4 in livers were detected by realtime RT-PCR. The data are normalized to *GAPDH*. *p<0.05, **p<0.01; N = 7–16 mice per group. The values were expressed as mean ± SD.

HMGB1 is shown to be increased during inflammation [13], and its receptors TLR2/TLR4 are demonstrated to be important in various types of obstructive cholangiopathy [18]. BA is a typical obstructive biliary disease with inflammation of extra and intra-hepatic bile ducts, so we investigated whether HMGB1 and TLRs were increased in livers from patients with BA. Livers derived from patients with congenital dilation of the bile duct (CDB), which were not ideal controls but with relatively minor liver lesion and easy to acquire due to ethical considerations [19], were used as controls to compare to BA livers. The clinical data of patients were summarized in **Table S1 and** described in **Supplemental Protocol S1**. Increased staining of HMGB1 in the periductal area and increased staining of TLR2 and TLR4 in the periductal area and infiltrated cells were noted in liver tissues of patients with BA compared to patients with CDB (**Fig. 1A**). Western blotting analyses showed significantly increased protein levels of HMGB1, TLR2 and TLR4 (all, *p*<0.05) in liver tissues from patients with BA compared to patients with CDB. The realtime RT-PCR showed the livers from patients with BA had significantly increased mRNA levels of HMGB1, TLR2 and TLR4 (all, *p*<0.05) compared to patients with CDB (**Fig. 1B and 1C**).

### Increased expression of HMGB1, TLR2 and TLR4 in the livers is observed in RRV-induced murine BA

To investigate whether murine BA has similar increases of HMGB1, TLR2 and TLR4 as human BA, we next detected their expression in livers of mice on day 7 after RRV-infection. The immunochemical staining showed that non-RRV infected wildtype (WT) C57BL/6 (B6) mouse pups (left panels in **Fig. 1D**) had low expression of HMGB1 mainly localized inside the nuclei of cholangiocytes. In pups exposed to RRV infection, increased HMGB1 staining was found not only in the mucosal layer of the cholangiocytes, but also in cells in the periductal areas. Realtime RT-PCR showed an approximately 7-fold increase of HMGB1 mRNA in the livers of RRV-infected pups compared to pups without RRV challenge (*p*<0.01) (**Fig. 1E**). Increased staining of TLR2 and TLR4 was observed in the livers of pups exposed to RRV infection (middle and right panels in **Fig. 1D**). Realtime RT-PCR showed that livers from RRV infected mice had a 4- to 6-fold increase of TLR2 and TLR4 compared to non-RRV challenged mice (both, *p*<0.01) (**Fig. 1F and 1G**). These findings confirmed that human BA and RRV-induced murine BA have similar increases of HMGB1, TLR2 and TLR4, indicating that murine BA, in part, mimics the pathophysiological changes of human BA.

### Rotavirus infection induces release of HMGB1 from cholangiocytes and macrophages

**Figure 2:**
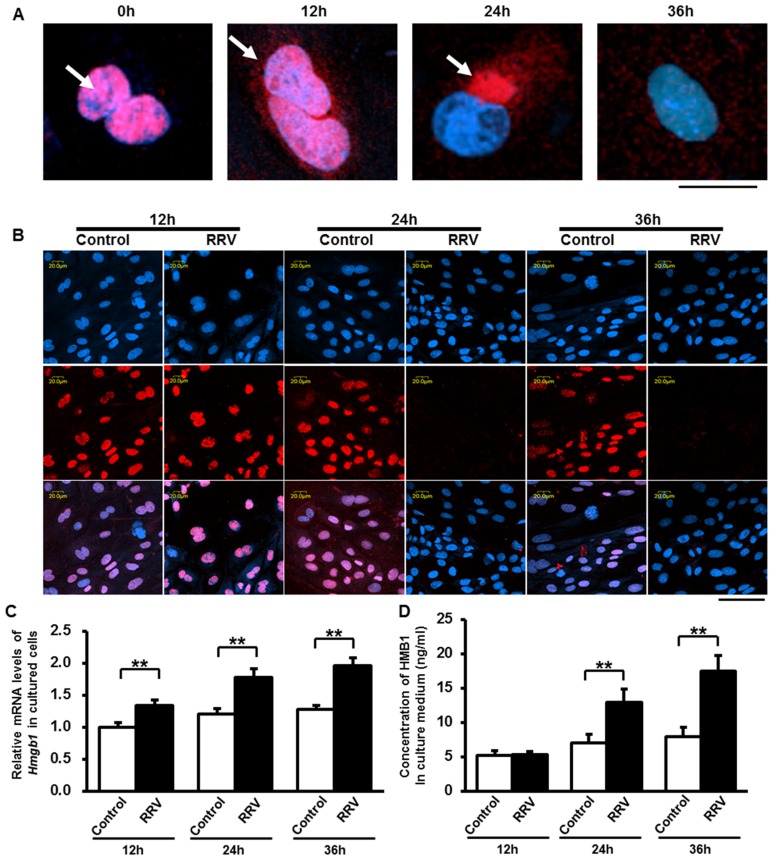
Synthesis and release of HMGB1 induced by RRV infection on cultured cholangiocytes. (**A**) Release of HMGB1 from the nuclei of cholangiocytes was detected after rhesus-rotavirus (RRV) infection at different time points by immunocytofluorescent staining. Representative images are showing the location relationship between HMGB1 (red) and nuclei (blue) at 0, 12, 24 and 36 hours after RRV infection. White arrows are showing the location of HMGB1 during release. The scale bar = 20 µm. (**B**) Immunocytofluorescent staining of HMGB1 in the nuclei of cultured cholangiocytes at 12, 24 and 36 hours after RRV infection. Cells in the control group were incubated with vehicle medium. The upper panels represent nuclei (blue), the middle panels represent HMGB1 staining (red), the lower panels represent overlays. The scale bar = 50 µm. (**C**) Quantification of the mRNA levels of HMGB1 in cultured cholangiocytes at 12, 24 and 36 hours after RRV infection. The values were normalized to *GAPDH*. (**D**) Concentration of released HMGB1 in the culture medium at 12, 24 and 36 hours after RRV infection. Four independent samples are tested in each group and each sample is run in triplicate. **p<0.01. The values were expressed as mean ± SD.

Now that we have confirmed the similarly increase of HMGB1 is noted bile ducts and periductal area both in human and murine BA, we designed *in vitro* studies to investigate whether RRV-infected cholangiocytes or macrophages release HMGB1. Immunofluorescent staining showed that HMGB1 was localized in nuclei of cholangiocytes at a 0 hour time point of RRV infection. HMGB1 release from nuclei began 12 hours after RRV incubation, and a large amount of nuclear HMGB1 in the nuclei was released extracellularly at 24 and 36 hours after RRV infection (**Fig. 2A**). Nuclear HMGB1 staining weakened starting from 24 hours after RRV infection, while HMGB1 in non-infected cholangiocytes was localized in the nuclei at all time points (**Fig. 2B**). The mRNA level of HMGB1 in cholangiocytes was increased significantly at 12 hours, 24 hours and 36 hours (all, *p*<0.01) after RRV infection compared to control groups (**Fig. 2C**). Unlike the mRNA levels, there was no significant difference in the protein level of HMGB1 in the culture medium between the infected and control cholangiocytes at the 12 hour time point (*p*>0.05), whereas the concentration of extracellular HMGB1 protein was increased significantly in the RRV-infected group compared to the control at the 24 hour and the 36 hour (both, *p*<0.01) time points. Although synthesis and release of HMGB1 were both increased, the percentage increase of HMGB1 release was higher than that of HMGB1 synthesis at 24 hour (84.4% *vs.* 47.4%) and 36 hour (119.8% *vs.* 54.0%) time points. This may cause decreased staining of HMGB1 in nuclei at 24 hour and 36 hour time points (**Fig. 2D**). In addition, 24 hours after RRV infection, both newborn and adult macrophages have increased release of HMGB1 compared to their controls respectively (both, *p*<0.05), however, there was no significant difference in the level of HMGB1 between the newborn and adult macrophages after RRV infection (*p*>0.05) (**Fig. S1**). These findings indicate that an important component of increased HMGB1 in RRV-induced murine BA may be the nuclei of RRV-infected cholangiocytes, as well as other RRV infected immunocytes such as macrophages; the increase of HMGB1 mRNA synthesis in cholangiocytes starts at an early time point, followed by persistent release of HMGB1 from 24 hours up to 36 hours after RRV infection.

### HMGB1 induces activation of NK cells as mice age from 1 day old mice to adult mice

**Figure 3:**
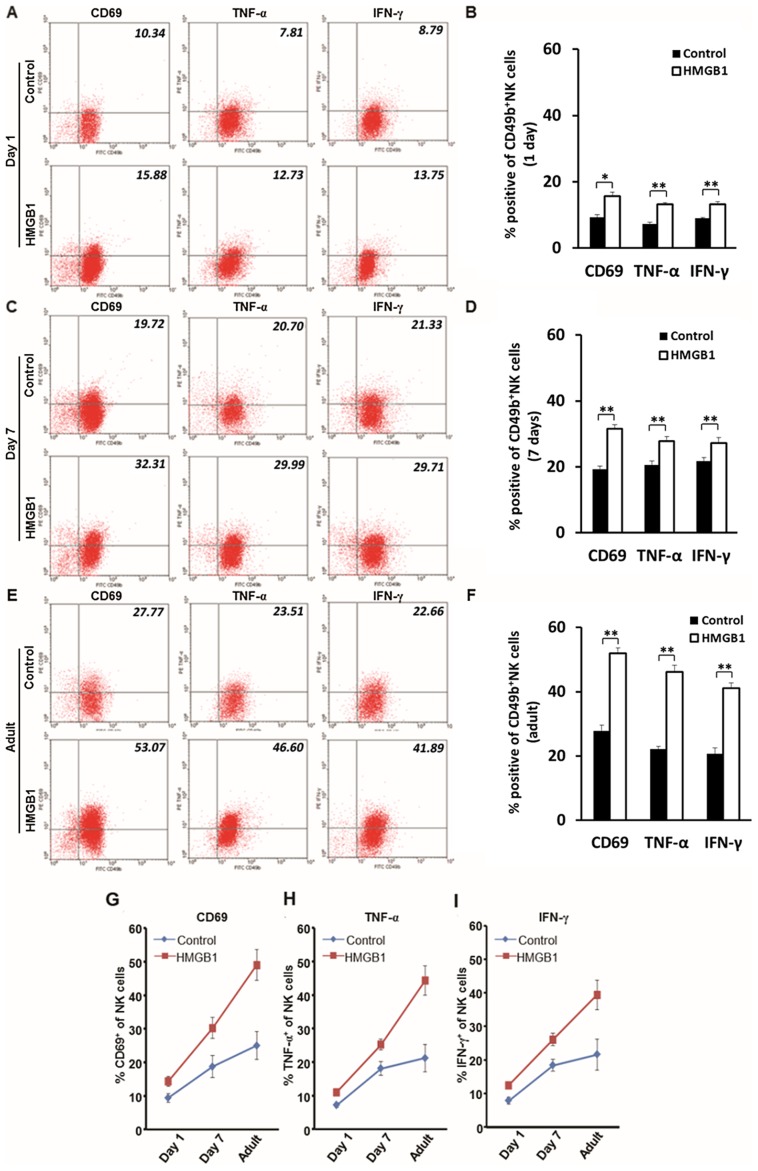
Age affects the activation of NK cells *in vitro*. (**A**, **C** and **E**) Flow cytometric analyses of activation markers of CD69, TNF-α and IFN-γ on CD49b^+^ NK cells stimulated by HMGB1 in different age groups. All NK cells were harvested from livers of mice of various ages. NK cells were either stimulated by HMGB1 or un-stimulated. Values in the right-upper quadrant of dot plots represent percent cells positive for CD49b and activation markers (CD69, TNF-α or IFN-γ) of NK cells, and the data are shown as representative dot plots. The average percentages of activation marker positive NK cells are shown in **B**, **D** and **F**. The change of percentages of CD69^+^ (**G**), TNF-α^+^ (**H**) and IFN-γ^+^ (**I**) NK cells in 1 day old, 7 day old and adult mice were illustrated in line charts. *p<0.05 and **p<0.01; N = 5 mice per group. The values are expressed as mean ± SD.

Our current studies have shown that an increased level of HMGB1 is observed in the development of BA, and NK cells are shown to play important roles in this disease [16], [17], we next investigated whether HMGB1 directly activates NK cells in a time-dependent fashion. The adult mice had the highest expression of CD69, TNF-α and IFN-γ compared to those in the other 2 age groups. We found significantly increased expression of CD69, TNF-α and IFN-γ in NK cells derived from all age groups in HMGB1-stimulated NK cells compared to non-stimulated NK cells (*p*<0.05 or *p*<0.01) (**Fig. 3A to 3F**). Additionally, compared to un-stimulated NK cells, HMGB1 stimulation of NK cells derived from adult mice increased the expression of CD69 from (27.70±1.88)% to (51.92±1.69)%, TNF-α from (22.13±0.81)% to (46.12±2.08)%, and IFN-γ from (22.66±0.47)% to (41.05±1.79)%; however, HMGB1 stimulation of NK cells derived from 7 day old mice only increased the expression of CD69 from (19.28±0.88)% to (31.61±1.22)%, TNF-α from (20.58±1.16)% to (27.77±1.45)% and IFN-γ from (21.69±1.08)% to (28.08±1.57)%, and HMGB1 stimulation on NK cells derived from 1 day old mice only increased the expression of CD69 from (9.33±0.73)% to (15.67±1.16)%, TNF-α from (7.31±0.49)% to (13.18±0.0.54)% and IFN-γ from (8.96±0.29)% to (13.17±0.84)% (**Fig. 3G to 3I**). These observations suggest that newborn NK cells have a poor response to HMGB1 stimulation, whereas HMGB1 stimulation induces higher activation of NK cells derived from older mice.

### Blockade of HMGB1 in NK cells induces decreased activation of NK cells

To analyze whether release of HMGB1 from RRV-infected cholangiocytes directly induces activation of NK cells, we performed co-culture of cholangiocytes (−/+RRV) and NK cells (−/+ HMGB1 −/+ anti-HMGB1 antibody), and investigated the expression of TNF-α and IFN-γ of NK cells by flow cytometric analyses. After RRV-infection in cholangiocytes or HMGB1 stimulation of NK cells, increased activation was noted in NK cells compared to controls (*p*<0.05 or *p*<0.01). NK cells pretreated with HMGB1 gained the highest expression of TNF-α and IFN-γ when cholangiocytes were infected with RRV. Additionally, when NK cells were incubated by the anti-HMGB1 antibody prior to co-culture, the expression of TNF-α and IFN-γ significantly decreased (*p*<0.05 or *p*<0.01) (**Fig. S2**). This evidence links the fact that HMGB1 released from RRV-infected cholangiocytes is one important source promoting the activation of NK cells.

### HMGB1 induces activation of NK cells via TLR2 and TLR4

**Figure 4:**
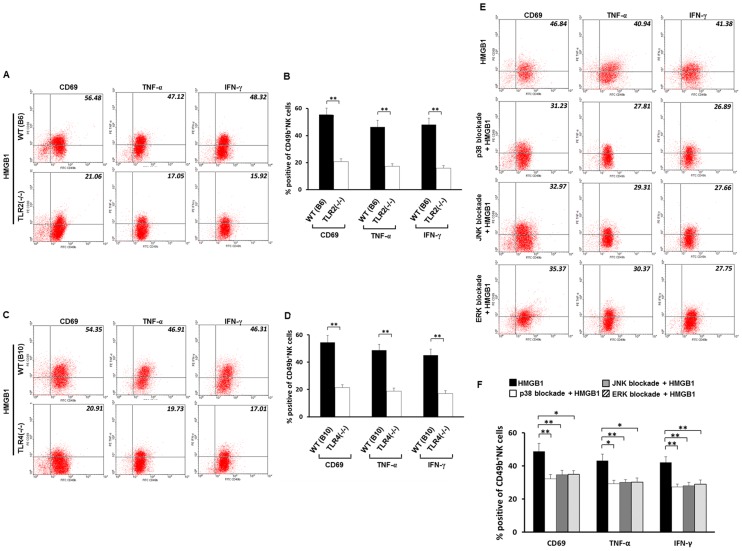
Roles of TLR2, TLR4 and MAPK families in HMGB1-induced activation of NK cells. (**A** and **C**) Flow cytometric analyses of activation markers on *Tlr2* ^−/−^ and *Tlr4* ^−/−^ NK cells (CD49b^+^) stimulated by HMGB1. NK cells were derived from livers of adult *Tlr2* ^−/−^ mice, *Tlr4* ^−/−^ mice and their wild-type controls. All NK cells in this experiment were stimulated by HMGB1. Data are shown as representative dot plots and the values in the right-upper quadrant represent percent cells positive for CD49b and activation markers of NK cells, and the average percentages of activation marker positive NK cells are shown in **B** and **D**. (**E**) Flow cytometric analyses of activation markers on HMGB1 stimulated CD49b^+^ NK cells under blockade of p38, JNK or ERK. All NK cells were derived from adult wild-type B6 mice. Values in the right-upper quadrant represent percent cells positive for CD49b and activation markers of NK cells and the average percentages of activation-marker positive NK cells are shown in **F**. *p<0.05, **p<0.01; N = 5 mice per group. The values are expressed as mean ± SD.

Because HMGB1 interacts with TLRs on various cell types [14], we next investigated whether HMGB1 activates NK cells via TLRs. We tested the effect of HMGB1 on NK cells derived from WT, *Tlr2* ^−/−^ and *Tlr4* ^−/−^ mice. HMGB1 stimulated NK cells derived from adult *Tlr2* ^−/−^ mice had decreased expression of CD69, TNF-α and IFN-γ (all, *p*<0.01) compared to HMGB1 stimulated NK cells derived from WT B6 controls (**Fig. 4A and 4B**). Similarly, HMGB1 stimulated NK cells derived from *Tlr4* ^−/−^ mice had decreased expression of CD69, TNF-α and IFN-γ (all, *p*<0.01) compared to HMGB1 stimulated NK cells derived from WT B10 controls (**Fig. 4C and 4D**). This evidence further indicates that TLR2 and TLR4 are both important in HMGB1-induced NK cell activation. Either TLR2 or TLR4 loss of function results in inadequate responses to HMGB1 stimulation, suggesting that HMGB1 stimulates NK cells via TLR2 and TLR4.

### HMGB1 activates NK cells via MAPK signaling pathways

Because MAPK activation is important in the TLRs-mediated upregulation of cytotoxicity and cytokine production in NK cells [20], we then explored whether NK cells are activated by HMGB1 via MAPK signaling pathways. Significantly decreased expression of CD69 (*p*<0.01), TNF-α (*p*<0.05) and IFN-γ (*p*<0.01) was noted in HMGB1 stimulated NK cells after p38 blockade compared to NK cells stimulated by HMGB1 without p38 blockade. JNK blockade of NK cells stimulated by HMGB1 also led to significantly decreased expression of CD69, and IFN-γ (all, *p*<0.01) compared to NK cells without JNK blockade but stimulated by HMGB1. Similarly, blockade of ERK also significantly decreased expression of CD69 (*p*<0.05), TNF-α (*p*<0.05) and IFN-γ (*p*<0.01) in NK cells stimulated by HMGB1 compared to NK cells without ERK blockade but stimulated by HMGB1 (**Fig. 4E and 4F**). These observations demonstrate that HMGB1 activates NK cells via activation of MAPK signaling pathways, in which inhibition of p38, JNK or ERK may lead to inadequate response of NK cells to HMGB1 stimulation.

### HMGB1 activates NK cells via the HMGB1-TLR2/TLR4-MAPK signaling pathways

**Figure 5:**
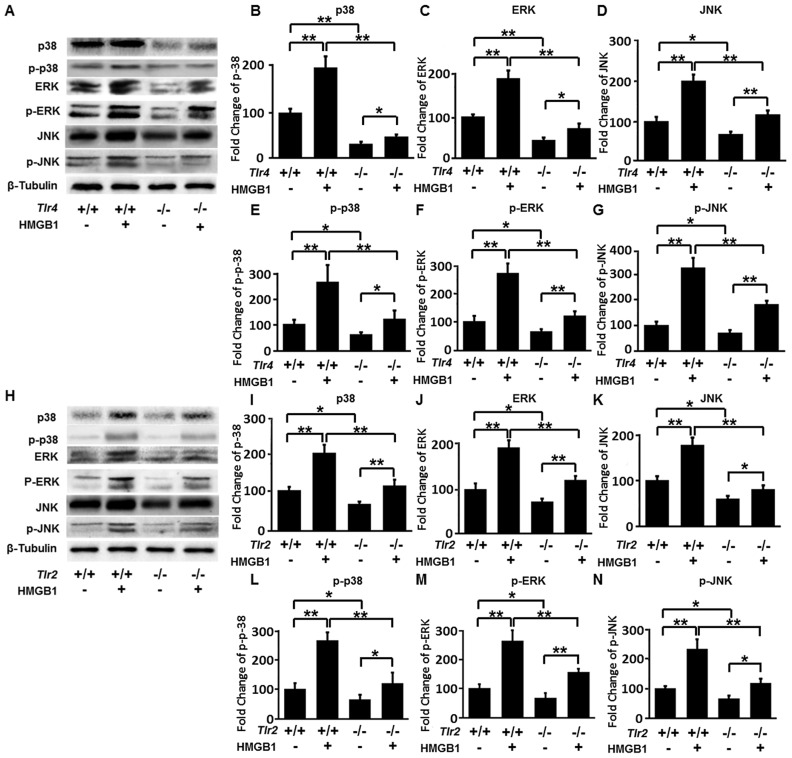
Expression of total and phosphorylated p38, ERK and JNK. (**A**) Specific bands of p38, p-p38, ERK, p-ERK, JNK and p-JNK of NK cells derived from adult wild-type B10 mice and *Tlr4* ^−/−^ mice were detected. NK cells were stimulated with HMGB1 or un-stimulated. (**B**–**G**) Fold changes of total and phosphorylated p38, ERK and JNK of NK cells derived from wild-type B10 mice and *Tlr4* ^−/−^ mice were quantified. (**H**) Specific bands of p38, p-p38, ERK, p-ERK, JNK and p-JNK of NK cells derived from adult wild-type B6 mice and *Tlr2* ^−/−^ mice were detected. (**I**–**N**) Relative protein levels of total and phosphorylated p38, ERK and JNK of NK cells derived from wild-type B6 mice and *Tlr2* ^−/−^ mice were quantified. *p<0.05, **p<0.01; N = 5 mice per group. The protein levels are normalized to internal β-Tubulin controls and expressed as mean ± SD.

Our findings have confirmed that HMGB1 activated NK cells via TLRs and MAPK signalings, we then tested whether NK cells in adult mice are activated via HMGB1-TLRs-MAPK signaling pathways. Without HMGB1 stimulation, WT mice had significantly higher expression of total and phosphorylated p38, ERK and JNK in NK cells compared to *Tlr4* ^−/−^ mice and *Tlr2* ^−/−^ mice (all, *p*<0.05 or *p*<0.01). When HMGB1 stimulation was performed, WT mice still had significantly higher expression of total and phosphorylated p38, ERK and JNK in NK cells compared to *Tlr4* ^−/−^ mice and *Tlr2* ^−/−^ mice (all, *p*<0.01) (**Fig. 5**). These data suggest that HMGB1 increases activation of MAPK signaling pathways in NK cells via TLRs. With absence of TLR4 or TLR2, HMGB1 can still activate MAPK signaling pathways in NK cells via TLR2 in *Tlr4* ^−/−^ mice or TLR4 in *Tlr2* ^−/−^ mice, however this activation level is lower than that in NK cells of WT mice. In addition, HMGB1 stimulation of NK cells derived from WT mice resulted in higher folder changes in phosphorylated protein levels compared to those in the total protein levels. Specifically, the total p38, ERK and JNK were increased to 1.99, 1.91 and 2.02 fold higher respectively compared to controls, while the p-p38, p-ERK and p-JNK were increased to 2.58, 2.63 and 3.18 folder higher respectively compared to controls. Similarly, HMGB1 stimulation of NK cells derived from TLR2(−/−) mice and TLR4(−/−) mice also led to higher, but more slighter, folder changes in phosphorylated protein levels compared to those in the total protein levels (**Fig. 5**). These data suggest that HMGB1 not only increases expression of MAPK families, but also directly increases phosphorylation of MAPK families in NK cells via TLR2 and TLR4, leading to effective and prompt activation of NK cells via HMGB1-TLR2/TLR4-MAPK signaling pathways.

### Inadequate activation of NK cells by HMGB1 is associated with low expression of TLRs on NK cells in newborn mice, and NK cells gain increased expression of TLRs gradually as mice age

We have shown that HMGB1 activates NK cells via TLR2 and TLR4, and a recent study has demonstrated that neonatal TLR-mediated responses are weaker compared to those of adults [21]. Due to these findings, we next investigated whether HMGB1 promotes the expression of TLR2 and TLR4 of NK cells in an age-dependent fashion.

**Figure 6:**
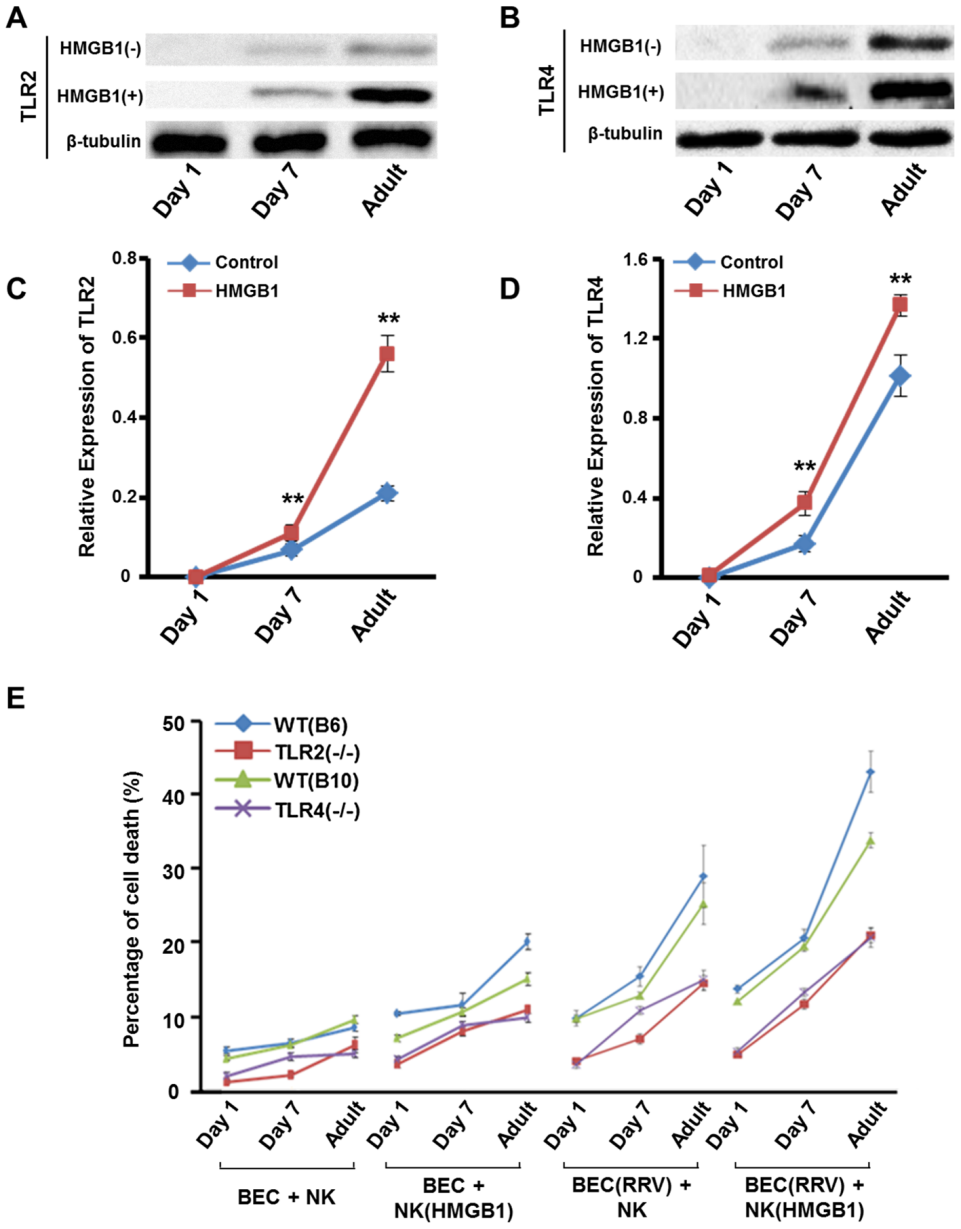
Expression of TLR2/TLR4 and NK cell cytotoxicity increases as mice age. (**A**) Specific bands of TLR2 of NK cells derived from *Tlr4* ^−/−^ mice and (**B**) specific bands of TLR4 of NK cells derived from *Tlr2* ^−/−^ mice with different ages (1 day, 7 days and 10 weeks). NK cells were stimulated by HMGB1 or un-stimulated. (**C**) The relative protein level of TLR2 of NK cells derived from *Tlr4* ^−/−^ mice in different age groups. **p<0.01 compared to the control group; N = 5 mice per group. The values are normalized to the expression of β-Tublin and expressed as mean ± SD. (**D**) The relative protein level of TLR2 of NK cells derived from *Tlr4* ^−/−^ mice in different age groups. **p<0.01 compared to the control group; N = 5 mice per group. The values are normalized to the expression of β-Tublin and expressed as mean ± SD. (**E**) Cytotoxicity is measured by percentage of cholangiocyte death. NK cells were derived from *Tlr2* ^−/−^ mice, *Tlr4* ^−/−^ mice and their wild-type controls. Livers of mice with different ages (1 day, 7 days and 10 weeks) were used as the source of NK cells. From the left to the right, the treatment was (1) non-RRV infected cholangiocytes + non-HMGB1 stimulated NK cells, (2) non-RRV infected cholangiocytes + non-HMGB1 stimulated NK cells, (3) RRV-infected cholangiocytes + non-HMGB1 stimulated NK cells or (4) RRV-infected cholangiocytes + HMGB1 stimulated NK cells. N = 5 mice per group. The values represent the percentages of cholangiocyte death and are expressed as mean ± SD.

Without HMGB1 stimulation, the protein levels of TLR2 and TLR4 of NK cells were extremely low in newborn mice. After 7 days of life, the mice had significantly increased expression of TLR2 and TLR4 (both, *p*<0.01) of NK cells compared to newborn mice. Moreover, the levels of TLR2 and TLR4 in adult mice were significantly higher than those in 7 day old mice (both, *p*<0.01). With HMGB1 stimulation, the expression of TLRs was increased significantly in 7 day old mice (TLR2, 0.110±0.021 *vs.* 0.067±0.016, *p*<0.01; TLR4, 0.373±0.060 *vs.* 0.170±0.040, *p*<0.01) and adult mice (TLR2, 0.560±0.046 vs. 0.211±0.020, *p*<0.01; TLR4, 1.367±1.013, *p*<0.01). Moreover, HMGB1-induced upregulation of TLR2 and TLR4 of NK cells in newborn mice was less significant than those in 7 day old mice and adult mice (**Fig. 6A to 6D**). These data suggest that the expression of TLR2 and TLR4 of NK cells increases in an age-dependent fashion.

### NK cells in newborn mice have very low cytotoxicity which increases gradually as mice age, and RRV infected cholangiocytes are more vulnerable to HMGB1-stimulated NK cell attack

Now that we have confirmed that HMGB1 activates NK cells in a time-dependent fashion, we next investigated whether NK cell cytotoxicity increases as mice age and whether HMGB1 stimulated NK cells have higher cytotoxicity on RRV infected cholangiocytes. We performed the NK-cell cytotoxicity assay based on our previous study [22]. NK cells derived from WT mice had persistently higher cytotoxicity than *Tlr2* ^−/−^ mice and *Tlr4* ^−/−^ mice in all age groups. Newborn NK cells derived from 1 dayold mice had the lowest cytotoxicity; while NK cells gained increasing cytotoxicity as mice aged, with the highest cytotoxicity seen in NK cells derived from adult mice. In the groups using RRV-infected cholangiocytes and HMGB1 stimulated NK cells, cytotoxicity of NK cells derived from WT mice increased approximately 3- to 3.5-fold in adult groups compared to 1 day old groups, and the cytotoxicity of NK cells derived from *Tlr2* ^−/−^ mice or *Tlr4* ^−/−^ mice was also increased in adult groups compared to 1 day old groups. Notably, the maximally cytotoxic effect was seen in HMGB1 stimulated NK cells interacting with RRV infected cholangiocytes in all 4 strains of mice (**Fig. 6E**). This experiment shows that TLR2 and TLR4 also take part in cytotoxicity of NK cells and newborn NK cells do not have sufficient cytotoxicity on the RRV-infected cholangiocytes.

Because a previous study [16] have shown that NK cells from livers of RRV-challenged mice are highly activated and are fully capable of killing cholangiocytes during the active phase of BA, and *in vivo* infection with RRV may reprogram the hepatobiliary immune response with effects on several different immune cell populations, we compared the *in vivo* NK cell cytotoxicity on cholangiocytes between NK cells from RRV-challenged neonatal mice and the NK cells from RRV-challenged adult mice. Results showed that 3 days after RRV infection, cytotoxicity of NK cells derived from RRV-infected adult mice increased significantly (*p<0.05), but subsequently decreased close to normal levels. RRV infection persistently increased the cytotoxicity of newborn mice after RRV infection as mice age (*p<0.05) (**Fig. S3**).

### RRV induces activation of NK cells *in vivo* in an age-dependent fashion

Our findings have shown that RRV-infected cholangiocytes release HMGB1 and that HMGB1 induces increasing activation of NK cells *in vitro* as mice age. We further confirmed that RRV-infection induces increasing activation of NK cells in an age-dependent fashion, by performing an *in vivo* experiment using a RRV-induced murine model of BA (**Fig. S4**). These data show that NK cells from newborn mice have a very limited response to RRV infection, whereas RRV challenge induces higher activation of NK cells in older mice, with the highest activation of NK cells seen in adult mice challenged with RRV.

### The incidence of BA and the level of VP4 in cholangiocytes are decreased as the age of mice increases

**Figure 7:**
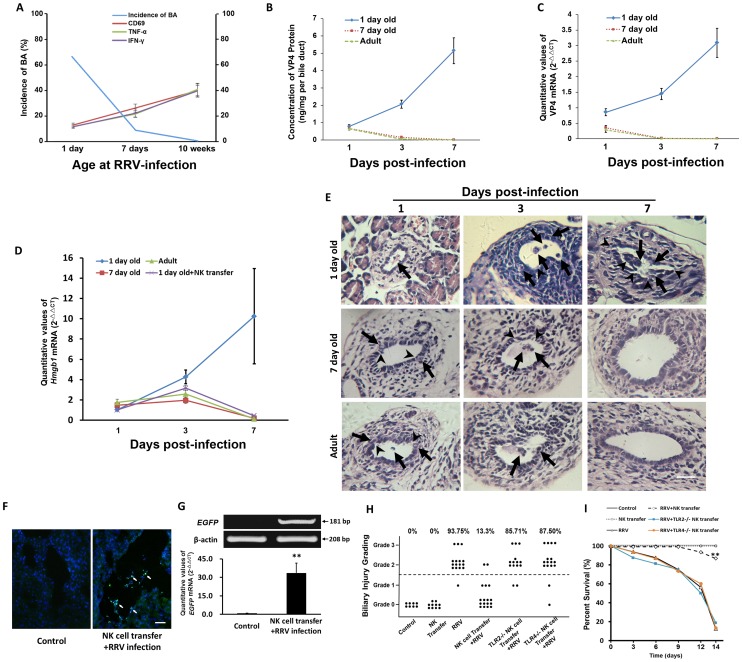
Adoptive transfer of mature NK cells decreases the incidence of BA and improves survival, and the level of VP4 in cholangiocytes and the incidence of BA are decreased as the age of mice increases. (**A**) The expression of CD69, TNF-α and IFN-γ of NK cells derived from the livers of B6 mice in different age groups was detected by flow cytometry (N = 5) at 24 hours after RRV challenge and the analysis of incidence of BA (N = 11–16). (**B** and **C**) RRV challenge was performed in mice of different age groups (N = 5). The protein and mRNA levels of VP4 in the bile ducts were detected by ELISA and realtime RT-PCR at different time points. The values of protein were expressed as mean ng/mg per bile duct weight ± SD. (**D**) The mRNA levels of HMGB1 in the bile ducts (N = 5 per group) were detected by realtime RT-PCR and are normalized to *GAPDH*. (**E**) Histopathological changes of bile ducts in different age groups on different days post-infection. The arrows indicate infiltrated inflammatory cells. The arrow heads indicate injured cholangiocytes. The scale bar = 50 µm. (**F**) Transferred EGFP-NK cells (white arrows) were found in the liver of recipient newborn B6 mice infected by RRV. The scale bar = 30 µm. (**G**) The upper panels show gel images representing EGFP mRNA levels in the livers by RT-PCR. The lower panel shows quantitative analysis of EGFP mRNA by realtime RT-PCR. **p<0.01. The data of mRNA levels are normalized to *β-actin*. (**H**) The summary of distribution of biliary injury grading. Each dot represents an individual mouse pup. The scale bar = 50 µm. (**I**) Survival analysis of mice subjected to different treatment (N = 8–16). **p<0.01.

To further confirm the role of the maturation of NK cells in the prevention of BA in mice, we compared the incidence of BA in different age group. When mice were infected by RRV on day 1 post-partum, 68.75% of mice developed BA 7 days after RRV infection; whereas only 9.1% of mice developed BA when mice were infected on day 7 post-partum; furthermore, no adult mice developed murine BA after RRV infection. These observations suggest that while RRV infection leads to increasing NK cell function as mice age, and these RRV-infected cholangiocytes may be eliminated by mature NK cells in time. Therefore, the incidence of BA decreases as the age at which mice are infected increases (**Fig. 7A**). To test whether mature NK cells can eliminate the RRV-infected cholangiocytes and prevent the continuous HMGB1 release, we then detected the levels of VP4, which represents the existence and maturity of RRV [23], and tested the levels of HMGB1 at different time points in mice with different ages after RRV infection. In the newborn group, ELISA and RT-PCR showed that the protein and mRNA levels of VP4 both increased gradually; while in the 7 day group and adult group, the protein and mRNA levels were detected on day 1 post-infection. However, these levels continued to decrease from day 1 through day 7(**Fig. 7B and 7C**). The mRNA level of HMGB1 in the newborn group increased from day 1 through day 7; while the mRNA level of HMGB1 was increased by approximately 30% in the 7 day group and by 50% in the adult group on day 3 compared to those on day 1 post-infection. However, the HMGB1 mRNA was decreased to undetectable levels in both groups on day 7 post-infection (**Fig. 7D**). Histopathological examination showed that after RRV infection, very few infiltrated inflammatory cells were observed in bile ducts with intact structure on day 1 post-infection. Increased inflammatory cells were observed in the bile duct lumens and submucosal layers on day 3 and through day 7 post-infection, with severely damaged cholangiocytes and obstructed lumens. In the 7 day old group and adult group, on day 1 and 3 post-infection, some damaged cholangiocytes were observed in the mucosal layers, beneath which inflammatory cells were observed in the submucosal layers. On day 7 post-infection, mice in these 2 groups had no infiltrated inflammatory cells, with intact biliary mucosal layers and unobstructed lumens (**Fig. 7E**). These results show that the adult mice avoid persistent RRV infection, continuous release of HMGB1 and persistent inflammation seen in newborn mice.

### Adoptive transfer of mature NK cells prior to RRV inoculation decreases the incidence of murine BA and improves survival of pups exposed to RRV infection

Having shown that immaturity of NK cells in newborn mice is important in the development of murine BA, we transferred NK cells derived from mature mice to newborn mice to test whether adoptive transfer of mature NK cells decreases the incidence of murine BA and improves survival of newborn pups. In order to track whether adoptively transferred NK cells could infiltrate in portal areas of livers of newborn recipient mice exposed to RRV infection, mature NK cells separated from the livers of adult EGFP mice were injected intraperitoneally to newborn B6 mice within 24 h after birth, prior to RRV infection. Before examining the effects of adoptively transferred NK cells on newborn mice infected by RRV, we first investigated how long the transferred NK cells persisted within the liver. One day after RRV infection plus NK cell transfer, NK cells were observed in the liver. On day 3 post-transfer, significantly increased NK cells were observed compared to those on day 1 (*p*<0.01). On day 7 post-transfer, the number of NK cells was significantly higher than that on day 3 (*p*<0.05). However, on day 14 post-transfer, the number of NK cells decreased significantly to a lower level (*p*<0.05) compared to that on day 7 (**Fig. S5**). Additionally, plaque forming assay (PFA) was performed within 24 hours of RRV infection after harvesting bile ducts from mice according to the method as described in our previous study [12]. The viral titer in the homogenized bile duct was (8.95±1.40)×10^3^ PFU/ml, and NK cell transfer prior to RRV infection did not decrease the yield of RRV in bile ducts (*p*>0.05); while in the control group (non-infected mice), the viral titer is 0 (**Fig. S6**). Twenty-four hours after adoptive transfer, we found that EGFP-NK cells were attaching to RRV-VP4 positive cells in biliary tree (white arrows indicating the site of NK cell attachment to RRV infected cells) (**Fig. S7**). Seventy-two hours after adoptive transfer, EGFP NK cells were observed in the portal areas of livers (**Fig. 7F**). No EGFP mRNA was detected in mice without EGFP-NK cell transfer, while a representative band (181 bp) was found in mice infected with RRV plus EGFP-NK cell transfer. Realtime RT-PCR analysis indicated that the mRNA level of EGFP mRNA in the livers of mice infected with RRV plus NK cell transfer was 33.61-fold higher than that of mice without NK cell transfer (*p*<0.01) (**Fig. 7G**). NK cell transfer in newborn mice led to decreased mRNA level of HMGB1 compared to that of the control on day 7 post RRV-infection (**Fig. 7D**). Fourteen days after RRV infection, 93.75% of mice exposed to RRV infection developed BA, whereas the adoptive transfer of 3×10^6^ liver NK cells significantly decreased the incidence of murine BA after RRV infection to 13.3% (*p*<0.01) (**Fig. 7H**). No mice in the control groups died after 14 days of life. However, only 12.5% mice survived after RRV infection, and adoptive transfer of NK cells improved survival significantly by 74.17% (*p*<0.01) (**Fig. 7I**). Additionally, when *Tlr2* or *Tlr4* deficient NK cells were transferred to newborn mice infected by RRV, they did not help decrease the incidence or improve the survival of BA, indicating that loss of *Tlr2* or *Tlr4* decreases the functions of NK cells derived from adult mice. This experiment shows that the maturation and normal functions of NK cells prevent mice from development of BA (**Fig. 7H and 7I**).

## Discussion

For the first time, we have demonstrated that HMGB1, TLR2 and TLR4 are over-expressed in liver tissues of infants with BA. We have also confirmed that RRV-infected cholangiocytes secrete HMGB1, which stimulates NK cells via the activation of the TLRs-MAPK signaling pathways. Meanwhile, we have shown that the NK cells in neonatal mice have limited responses to eliminate RRV-infected cholangiocytes due to the immaturity of NK cells, which leads to persistent RRV infection in cholangiocytes before maturation of NK cells. As RRV-infected mice age, gradual maturation of NK cells leads to persistent destruction of RRV-infected cholangiocytes, which finally, in part, causes progressive damage to cholangiocytes and the development of murine BA (**Fig. S8**). Adoptive transfer of mature NK cells prior to RRV inoculation leads to decreased incidence of BA and improved survival. In addition, due to the increasing activation of NK cells, the number of RRV-infected cholangiocytes and incidence of BA decrease as the age of mice increases. The activation of NK cells of adult mice is increased after RRV infection, and these activated NK cells eliminate RRV-infected cholangiocytes shortly after infection, decrease the persistent RRV infection and continuous HMGB1 release. Thus, they prevent the development of BA in adult mice. Our studies provide solid evidence showing that the maturation of NK cells is critical during the immunological gap of murine BA, which may provide direction for future clinical investigations regarding the mechanism of BA in human patients.

Our current clinical investigation of BA confirmed the presence of over-expression of HMGB1, TLR2 and TLR4 in the periductal areas and infiltrated inflammatory cells. It has been shown that the levels of TLR2 and TLR4 on various cells are increased during the initiation and modulation of the immune response to rotavirus infection [24], [25], and that HMGB1 acts as a pro-inflammatory cytokine that causes persistent inflammatory responses via activation of TLR2 and TLR4 [26]. Therefore, from the increased levels of HMGB1 and its receptors (TLR2 and TLR4), strong inflammatory responses are expected in the bile ducts of human patients with BA. Notably, the murine BA in our study mimics the pathogenesis of human patients with BA as seen by increased TLR2 and TLR4 and overexpression of HMGB1 in bile ducts in both the murine model of BA and in human BA. This indicates that human BA and RRV-induced murine BA may have similar pathomechanisms during disease development.

Previous studies have demonstrated that increased release of HMGB1 triggers an initial immune reaction which causes persistent tissue damage [27]. Since our study also shows that HMGB1 is increased in both human and murine BA, it is necessary to clarify where the HMGB1 originates. Our *in vitro* studies show that HMGB1 is initially released from the nuclei of RRV-infected cholangiocytes, with increased release of HMGB1 observed in a time-dependent manner. In addition to cholangiocytes, the periductal area also had increased staining of HMGB1 (**Fig. 1**), which may not only be released from RRV-infected cholangiocytes, but may also come from many other immunocytes including RRV-infected macrophages which are considered to be the targets of RRV infection [28]. As shown in this study, both newborn and adult macrophages can release HMGB1 after 24 hours after RRV infection (**Fig. S1**). Thus, the RRV-infected cholangiocytes, besides many other immunocytes such as macrophages, is one critical source of HMGB1 in triggering the inflammatory responses after RRV infection.

NK cells have been shown the ability to mediate biliary injury in murine model of BA in Shivakumar et al's study, in which the researchers have demonstrated that neonatal NK cells target the mouse bile duct epithelia via NKG2D and drive experimental BA [16]. They focused on rotavirus-primed hepatic NK cells attack and lysis of cholangiocytes in a contact- and NKG2D-dependent fashion and showed the presence of NkG2D ligands on cholangiocytes as well as the differential expression of these ligands on cells from diverse origin other than cholangiocytes. It is not yet proven whether HMGB1 directly stimulate NK cells via NKG2D, however, HMGB1 recognizes TLR2 and TLR4 [26]. We therefore explored the roles of TLR2 and TLR4 in HMGB1 mediated NK cell activation. We demonstrated that TLR2 or TLR4 loss of function caused greatly reduced HMGB-1 induced activation of NK cells by decreasing the expression of TNF-α, IFN-γ and CD69. It has been shown that NK cells respond to pathogens through TLR2 or TLR4 to produce pro-inflammatory cytokines such as TNF-α and IFN-γ [29], [30], and that these cytokines are important for host innate immune defense. All NK cell subsets express CD69, which is up-regulated after stimulation of TLR ligands or cytokines. This means that the level of CD69 on NK cells, representing the ability of NK cells to respond to pathogens, is regulated by activation of TLRs [31]. These studies support our observations that TLR2 and TLR4 are essential in the HMGB1-induced activation of NK cells. Because MAPK activation plays important roles in the TLRs-mediated up-regulation of cytotoxicity and cytokine production in NK cells [20], and HMGB1 is well known as a potent stimulating ligand of TLRs [26], it is reasonable to propose that HMGB1 activates the TLRs-mediated activation MAPK signaling pathways (p38/ERK/JNK). Combining others findings and our observations, there may be other cytokines or ligands that activate NK cells via the other signaling pathway inciting inflammatory responses, however HMGB1 is, at least, one of the cytokines secreted from injured cholangiocytes that can activate NK cells via TLR-MAPK signaling pathways.

In the current study, when newborn mice were infected with RRV, their cholangiocytes were infected by RRV shortly after RRV challenge. A previous study showed that after newborn mice were infected with RRV, the peak amount of RRV protein and live RRV in liver was detected around day 7 post-infection [32]. We have shown that in our model of BA, the level of VP4, which represents the presence of RRV, in the RRV-infected newborn mice continued to increase (**Fig. 7B and 7C**), which is solid evidence showing the presence of persistent RRV infection in the cholangiocytes. On the other hand, the clearance of VP4 by mature NK cells reflects the fact that mature NK cells lead to immediate elimination of RRV-infected cholangiocytes, which cuts off any further persistent RRV infection, subsequent biliary destruction or death of mice induced by RRV infection. Moreover, newborn NK cells have been shown to have low expression of TLR2 and TLR4 as well as low activation of MAPK families, thus they do not secrete sufficient TNF-α or IFN-γ, leading to low cytotoxicity of NK cells. Here we have shown that the activation of NK cells in newborn mice was only approximately 50% of that in 7 day old mice and only approximately 25% of that in adult mice (**Fig. 3**). Our findings were supported by Koo GC et al's study [33], in which they demonstrate that Nk-1.1 antigen as an early hemopoietic differentiation antigen, and Nk-1- cells could be induced by interferon to become NK-1+ cells, which activate NK cell cytotoxicity as mice age from fetuses to young adults. We speculate that this may be the rationale for the younger mice to have lower basal activation compared to adult mice. Thus, these newborn NK cells are incapable of attacking or eliminating RRV-infected cholangiocytes, and these cholangiocytes acquire persistent and gradually aggravated RRV infection for at least 7 days. During this period of RRV infection, with the maturation of NK cells and persistent release of HMGB1 as mice age (**Fig. 7D**), NK cells gradually increase expression of TLRs and MAPK families, thus their cytotoxicity increases as well. Along with the increasing release of HMGB1 in newborn mice infected by RRV, our histopathological findings confirmed progressively increased inflammation and aggregated injury in bile ducts (**Fig. 7E**), suggesting these matured NK cells attack RRV-infected cholangiocytes persistently and cause damage in bile ducts, which finally leads to development of murine BA (**Fig. S8**). In particular, our additional study shows that adoptive transfer of mature NK cells into newborn mice decreases biliary injury by more than 80% and improves survival of mice challenged with RRV by 74.17% (**Fig. 7H and 7I**). This is direct evidence strongly supporting our hypothesis that immaturity of NK cells in newborn mice is a crucial factor in the development of BA.

Our findings not only reveal a possible mechanism for the development of BA, but more importantly, can explain why newborn mice challenged with RRV have a very high incidence of BA, while older mice do not develop BA after RRV infection. This phenomenon is called the immunological gap of murine BA and was first described by Czech-Schmidt et al [15]. In their studies, they demonstrated that the highest incidence of BA was achieved by infection with RRV within the first 12 hours postpartum. However, the later the newborn mice were infected, the less likely they developed BA [15]. Since then, some studies have focused on the immunological gap and the triggering mechanisms that induce BA following rotavirus infection. Recently, Mohanty SK et al also reported an infection rate of 100% if injected at 24 hours with a mortality of 80% and 95% symptomatic mice, and a mortality of 95% if injected within 3 days after birth [34]. According to these reports, the “immunological gap” therefore may exist between days 1 and 3 reflecting the ability of the rotavirus to enter the cholangiocytes and reprogram the immune responses to a predominantly Th1 phenotype and initiate duct injury and immune cell driven duct pathogenesis. Recent studies have demonstrated that a post-natal paucity of regulatory T cells is important in the development of experimental BA [17] and that controls of Th1 cells [35], CD8^+^ T-lymphocytes [36] and NK cell activation are crucial in this disease [17]. We focused our studies on the maturation and activation of NK cells in murine BA because NK cells are the primary source of pro-inflammatory productions, such as IFN-γ and TNF-α, mere hours after infection occurs before the acquired immunity is established [37], [38], yet they are immature and dysfunctional in neonatal stages [39]. These studies combined with our present results strongly suggest that the expression of TLRs and activation of MAPK signaling pathways of NK cells increase gradually as mice age from neonates to adults. As a result, the cytotoxicity and expression of pro-inflammatory cytokines of NK cells grow from very low to adult levels. In our model of BA, as is mentioned above, the NK cells in newborn mice infected by RRV are incapable of attacking or eliminating RRV-infected cholangiocytes, which leads to persistent viral infection in their bile ducts, increasing release of HMGB1 and destruction of cholangiocytes when the NK cells maturate with the aging of these newborn mice with persistent RRV infection. To the contrary, in older mice infected by RRV, NK cell activation of 7 day old and adult mice is increased by approximately 100% and 200% respectively compared to that of newborn mice, and the protein level of VP4 in bile ducts of 7 day old and adult mice on day 3 post-infection is decreased by 92.1% and 97.4% respectively compared to that in newborn mice (**Fig. 3** **and Fig. S4**). Importantly, during the first 3 days post-infection, HMGB1 release slightly increases in older mice, which induces activation of NK cells in these older mice and helps eliminate RRV-infected cholangiocytes shortly after infection, which prevents any possibility of further persistent RRV infection in bile ducts. After the elimination of these infected cholangiocytes, the HMGB1 decreases to low levels, with the fading of inflammation and injury in bile ducts confirmed by our histopathological findings (**Fig. 7D and 7E**). Therefore, neither the biliary tracts are damaged nor does BA develop in older mice. Thus, our findings answer the question of the immunological gap in murine BA that was raised more than a decade ago.

In summary, we have demonstrated that in RRV-induced murine BA, HMGB1, which is initially released from injured cholangiocytes, is an inflammatory initiator; and our findings that HMGB1 promotes maturation and activation of NK cells via the TLRs-MAPK signaling pathways and that the role of NK cell maturation in the immunological gap of BA provide new approaches into the mechanistic study of NK cell-mediated RRV-induced BA. In future, novel therapies targeting HMGB1 or TLRs in patients with BA may be applied to decrease the activity of NK cells in order to inhibit the progression of BA. Despite this, our finding may be only one of the many mechanisms to explain the development of BA. We need to notice the importance of the other factors which also contribute in the development of BA, for example the regulatory T cells play key roles in regulating the immune system and mediating the development of BA [17], [35], [36]. Additionally, the macrophages [28], [40], dendritic cells [41] and CD8^+^ T cells [7], [42], play different roles and control various links to mediate the development of BA. Our theory that HMGB1-promoted and TLR-dependent NK cell maturation and activation my take part in development of murine BA, along with many other forerunners' theories, contributes to revealing the mechanisms of this unique neonatal disease –BA.

## Materials and Methods

### Ethics statement

Informed consent forms of this study giving parents' permission were signed. The Institutional Review Boards of Tongji Medical College approved the protocol of the study (Permit Number 2009-HP0368, Wuhan, China), which complied with the Helsinki Declaration revised in 1983.

All of the animal studies were carried out in accordance with Chinese Council on Animal Care in an AAALAC-accredited facility following approval of study design (Permit Number 2009-AR0288 and 2010-AR0486) by the Institutional Animal Care and Use of the Committee at Tongji Medical College (Wuhan, China). Veterinarians skilled in the healthcare and maintenance of rodents supervised animal care. Reasonable efforts were made to minimize suffering of animals. The use of animals was minimized by using an experimental design permitting statistically-significant changes to be demonstrated with the smallest number of animals per group and the smallest number of groups, which was consistent with scientific rigor.

### Human liver tissues and clinical data

The acquisition and use of human liver tissues were approved by the Institutional Review Boards of Tongji Medical College (Permit Number 2009-HP0368, Wuhan, China). Written informed consent was obtained from the guardians of the patients. The information of patients was recorded in **Table S1**. Grouping and treatment of liver samples were described in the **Supplemental Protocol S1**.

### Animals

*Tlr2* ^−/−^ B6 mice, *Tlr4* ^−/−^ B10 mice and EGFP mice were purchased from the Jackson Laboratory (Bar Harbor, Maine, USA). Control B6 mice and B10 mice were purchased from the Laboratory Animal Center in Wuhan University (Wuhan, China). Mice were bred under specific pathogen-free conditions. All animal protocols (Permit Number 2009-AR0288 and 2010-AR0486) were carried out according to the guidelines of the Chinese Council on Animal Care and approved by the Institutional Animal Care and Use of the Committee at Tongji Medical College (Wuhan, China).

### Mouse model of BA

Experimental BA [12] was described in **Supplemental Protocol S1**. Mice presenting jaundice, dehydration or moribund were euthanized immediately and all survived mice were euthanized on day 7 or 14 after RRV injection.

### RRV infection on cultured cholangiocytes or macrophages

As described in our previous study [43], extrahepatic cholangiocytes were isolated, cultured and identified. The purified cholangiocytes were >95% pure [43]. The method of macrophage culture was described in **Supplemental Protocol S1**. On the 4th day of culture, the cholangiocytes or macrophages were incubated with 500 µl RRV supernatant (2.0×10^6^ Plaque Forming Unit/ml) as described in our previous study [12].

### Immunocytofluorescent staining of HMGB1

The cholangiocytes were incubated with rabbit anti-HMGB1 antibody diluted at 1∶100 (ab 18256, Abcam, Cambridge, Massachusetts, USA), followed by incubation with Rhodamine-conjugated goat-anti-rabbit secondary antibody (Millipore Corporation, Billerica, Massachusetts, USA). The nuclei were counterstained with 4′,6-Diamidino-2-Phenylindole Dihydrochloride (DAPI).

### ELISA

The concentration of HMGB1 in the culture medium was determined using the HMGB1 ELISA-kit (Shino-test Corporation, Kanagawa, Japan). The extrahepatic bile ducts of RRV-infected mice in different age groups were harvested, weighed and homogenized, and the concentration of VP4 (ng/mg per bile duct) of mice (N = 5) was detected by a sandwich ELISA (Corning Costar, Cambridge, Massachusetts, USA). The methods for ELISA were described in **Supplemental Protocol S1**.

### Real-time RT-PCR

Complimentary DNA was synthesized from total RNA using SuperScript First-Strand cDNA Synthesis system for RT-PCR kit(Invitrogen, Carlsbad, California, USA). Real-time RT-PCR was performed using SYBR-Green PCR Master-Mix (Applied-Biosystems, Foster City, California, USA). The fluorescent signals were detected using an ABI7700 Real-time Sequence-detection system (Applied-Biosystems, Foster City, California, USA). The primers (as listed in **Table S2**) are synthesized by Invitrogen (Invitrogen, Carlsbad, California, USA). All tests were run in triplicate. The values were normalized to *GAPDH* or *β-actin*.

### Pathohistological analysis and immunohistochemistry

Murine bile duct injury was graded as described in our previous studies [12]. Unstained paraffin sections were incubated with primary antibodies (as listed in **Table S3**). The methods for immunohistochemistry were described in **Supplemental Protocol S1**.

### RRV-infection in mice at different ages

Three groups of B6 mice with the age of 1 day, 7 days and 10 weeks were challenged with 25 µl RRV supernatant (2.0×10^6^ Plaque Forming Unit/ml) per gram of bodyweight intraperitoneally (N = 11–16 for each group). The controls were only challenged with vehicle culture medium. Twenty-four hours later, these mice were euthanized and the blood in their livers was cleared by the infusion of PBS through the portal vein. Additional mice (N = 5) in each group were euthanized on day 1, day 3 and day 7 post-infection for detection of VP4 protein and mRNA levels.

### Isolation and purification of hepatic NK cells

The hepatic NK cells were isolated and purified using magnetic cell sorting as described in our previous study [22]. The sorted cells were >95% pure and the viability was >95% by the trypan blue exclusion test [22].

### Blockade of NK cell signaling pathways

The isolated NK cells were pretreated with pretreated with 10 µmol/L of p38 inhibitor SB203580 (Cell Signaling Technology, Danvers, Massachusetts), 10 µmol/L of ERK1/2 inhibitor U0126 (Cell Signaling Technology, Danvers, Massachusetts) or 20 µmol/L of JNK1/2 inhibitor SP600125 (Calibiochem, San Diego, California, USA) for 2 h at 37°C. The NK cells in the non-blockade control groups were only pretreated with DMSO for 2 h at 37°C. After blockade, the NK cells were ready for HMGB1 stimulation.

### HMGB1 stimulation

HMGB1 stimulation on NK cells was described in the **Supplemental Protocol S1**.

### Flow cytometric analysis

Two-color flow cytometric analysis was performed. The NK cells were resuspended in a concentration of 1×10^7^ cells/ml. All cells were stained with FITC anti-mouse CD49b, followed by staining of PE anti-mouse CD69, PE anti-mouse IFN-γ or PE-anti-mouse TNF-α antibody (San Diego, California, USA). The methods were described in **Supplemental Protocol S1**.

### Western blotting analysis

Protein extracts were subjected to SDS-PAGE and the proteins were transferred to nitrocellulose membranes followed by antibody incubation (as listed in **Table S4**). The methods for western blotting were described in **Supplemental Protocol S1**.

### NK-cell cytotoxicity assay against cultured cholangiocytes

Grouping and methods for the cytotoxicity assay [44] of liver NK cells (effector cells) against cultured cholangiocytes (target cells) were described in **Supplemental Protocol S1**.

### Adoptive transfer of mature NK cells

Liver NK cells were derived from adult EGFP mice or B6 mice using magnetic cell sorting as described in our previous study [22]. Isolated NK cells (3×10^6^) in 50 µl PBS were transferred intraperitoneally into newborn B6 mice (N = 15) prior to RRV injection within 24 hours after birth; whereas in the control group, newborn B6 mice only received NK cells without RRV infection (N = 10). Another 2 groups of mice received only 50 µl PBS (N = 8) or 50 µl RRV supernatant (N = 16). Additionally, *Tlr2*−/− or *Tlr4*−/− NK cells were transferred in 2 groups of newborn B6 mice (N = 14 and N = 16 respectively). The pathophysiological changes were observed, and the incidence of BA and survival of mice were recorded. Liver samples were frozen sectioned and the fluorescence of transferred NK cells with green fluorescence was observed, and the EGFP mRNA levels in the livers were compared using RT-PCR and realtime RT-PCR. The β-actin was used as the internal control and the information of primers was listed in **Table S2**.

### Statistical analysis

Results, which were presented as mean value ± standard deviation (SD), were compared using Student's *t* test. For experiments with more than 2 groups, statistical comparisons between all groups were performed using one-way analysis of variance, and further comparison between each of 2 groups were performed using Student's *t* test. The incidence of BA and the survival rate were compared using Fisher's exact Test.

## Supporting Information

Figure S1 Rotavirus infection induces release of HMGB1 from macrophages. The concentration (ng/ml) of HMGB1 released from macrophages derived from newborn and adult mice was evaluated in the culture medium by ELISA and expressed as mean ± SD.
(TIF)

Figure S2 HMGB1 released from RRV-infected cholangiocytes promotes NK cell activation. (**A**) Flow cytometric analyses of expression of TNF-α and IFN-γ on CD49b^+^ NK cells stimulated by HMGB1. All NK cells were harvested from livers of adult wild-type B6 mice. The NK cells were treated with −/+ anti-HMGB1 antibody prior to −/+ HMGB1 stimulation, and the cholangiocytes were pretreated with −/+ RRV. Values in the right-upper quadrant of dot plots represent percent cells positive for CD49b and expression of TNF-α and IFN-γ of NK cells. The data are shown as representative dot plots. The average percentages of TNF-α^+^ and IFN-γ+ CD49b^+^NK cells are shown in **B** and **C**.
(TIF)

Figure S3 *In vivo* cytotoxicity assay of NK cells of RRV-infected mice at different ages. Cytotoxicity is measured by percentage of cholangiocyte death. NK cells were derived from newborn mice −/+ RRV infection or adult mice −/+ RRV infection. One day, 3 days and 7 days after RRV infection, livers of mice were used as the source of NK cells. N = 5 mice per group. The values represent the percentages of cholangiocyte death and are expressed as mean ± SD.
(TIF)

Figure S4 Age affects the RRV-induced activation of NK cells *in vivo.* (**A**, **C** and **E**) Flow cytometric analyses of activation markers of CD69, TNF-α and IFN-γ on CD49b^+^ NK cells in B6 mice challenged with RRV at different age groups (1 day old, 7 day old and 10 week old). Mice were injected with vehicle or RRV. NK cells were harvested from the livers of mice at 24 hours after RRV challenge. Values in the right-upper quadrant represent of dot plots percent cells positive for CD49b and activation markers of NK cells and the data are shown as representative dot plots. The average percentages of activation marker positive NK cells are shown in **B**, **D** and **F**. The change of percentages of CD69^+^ (**G**), TNF-α^+^ (**H**) and IFN-γ^+^ (**I**) NK cells and average percentage of activation marker positive NK cells in the 1 day, 7 day and adult groups was illustrated in line charts. **p<0.01; N = 5 mice per group. The values are expressed as mean ± SD.
(TIF)

Figure S5 Tracking of transferred NK cells in the liver. The transferred EGFP NK cells were tracked in the liver at 0, 1, 3, 7 and 14 days post-transfer (**A to E**). Green fluorescence represents EGFP NK cells and the DAPI is used for counterstaining of nuclei. N = 5 mice per group. The magnification is 200×. (**F**) The values represent the average of EGFP NK cells in the liver per vision field and are expressed as mean ± SD.
(TIF)

Figure S6 Plaque forming assay for detection of RRV in bile ducts. Viral titer in the control mice, RRV infected mice and RRV infected mice with prior NK cell transfer. The mean viral titer in the bile duct was expressed as mean±SD (10^3^ PFU/ml/mg).
(TIF)

Figure S7 NK cells attaching to RRV infected cells. The transferred EGFP NK cells (green fluorescence) were tracked in the liver 24 hours after NK cell transfer in non-infected mice (**A**) or RRV-infected mice (**B**). VP4 staining (red fluorescence) represents for RRV infected cells in the biliary tree. The white arrows indicate the sites where the NK cells attach the RRV infected cells.
(TIF)

Figure S8 The relationship between maturation of NK cells and development of BA. After RRV infects bile ducts of 1 day old newborn mice, the cholangiocytes begin to secrete HMGB1. However, due to the low expressions of TLR2 and TLR4 on newborn NK cells, HMGB1 is incapable of activating NK cells via TLRs-MAPK signaling pathway. Thus, neither enough pro-inflammatory cytokines are secreted, nor is there enough NK cell cytotoxicity to eliminate RRV injured cholangiocytes. These factors cause persistent RRV infection in cholangiocytes of newborn mice. As mice age, they gain increased expressions of TLR2 and TLR4 on their NK cells and the persistently infected cholangiocytes persistently release HMGB1. These matured NK cells are capable of recognizing the increased HMGB1 signal, leading to activation of NK cells via the TLRs-MAPK signaling pathway. Activated NK cells secrete large amounts of pro-inflammatory cytokines, and generate persistent cytotoxicity on RRV-infected cholangiocytes, thus the cholangiocytes are persistently damaged by pro-inflammatory cytokines and direct NK cell cytotoxicity. With destruction of extrahepatic bile duct lumens, mice challenged with RRV at their neonatal stage consequently develop BA. On the other hand, the activation of NK cells of adult mice is increased, thus their mature NK cells eliminate RRV-infected cholangiocytes shortly after infection, which prevents any possibility of persistent RRV infection in bile ducts and further release of HMGB1. Therefore, neither the biliary tracts are further damaged nor does BA develop.
(TIF)

Table S1 Clinical data of patients diagnosed with biliary atresia (BA) and congenital dilation of the bile duct (CDB).
(DOCX)

Table S2 Gene name and nucleotide composition for primers used in real-time RT PCR.
(DOCX)

Table S3 Antibodies for immunohistochemical or immunofluorescent staining.
(DOCX)

Table S4 Antibodies for western blotting.
(DOCX)

Supplemental Protocol S1 Human liver tissues and clinical data. Mouse model of BA. ELISA. Pathohistological analysis, immunohistochemistry and immunofluorescent staining. HMGB1 stimulation. Flow cytometric analysis. Co-culture of cholangiocytes and NK cells. Western blotting analysis. NK-cell cytotoxicity assay against cultured cholangiocytes.
(DOC)
