# Supplementary figures and images for: HMGB1-Promoted and TLR2/4-Dependent NK Cell Maturation and Activation Take Part in Rotavirus-Induced Murine Biliary Atresia
Source: PLoS Pathog. 2014 Mar 20;10(3):e1004011. doi: 10.1371/journal.ppat.1004011 (PMC3961347; doi:10.1371/journal.ppat.1004011)

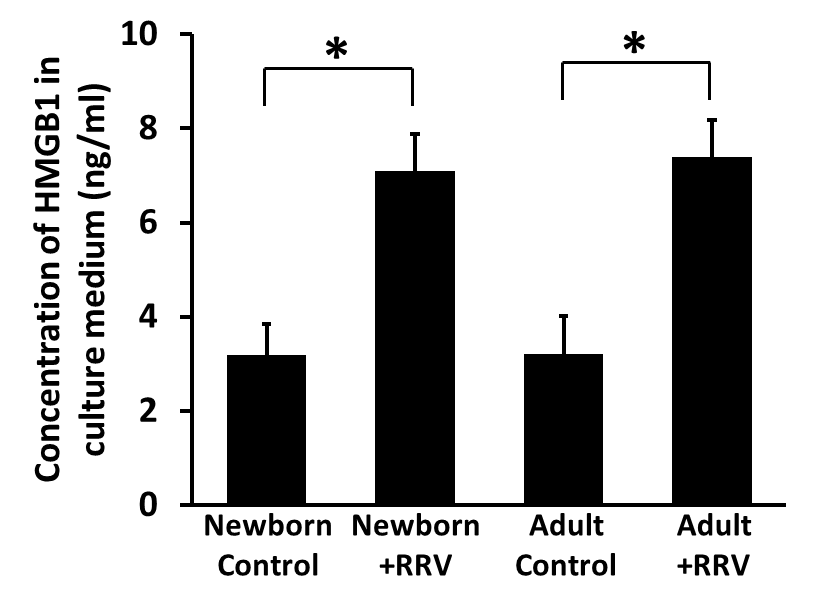

Supplement: Figure S1 — Rotavirus infection induces release of HMGB1 from macrophages. The concentration (ng/ml) of HMGB1 released from macrophages derived from newborn and adult mice was evaluated in the culture medium by ELISA and expressed as mean ± SD. (TIF) [file ppat.1004011.s001.tif]

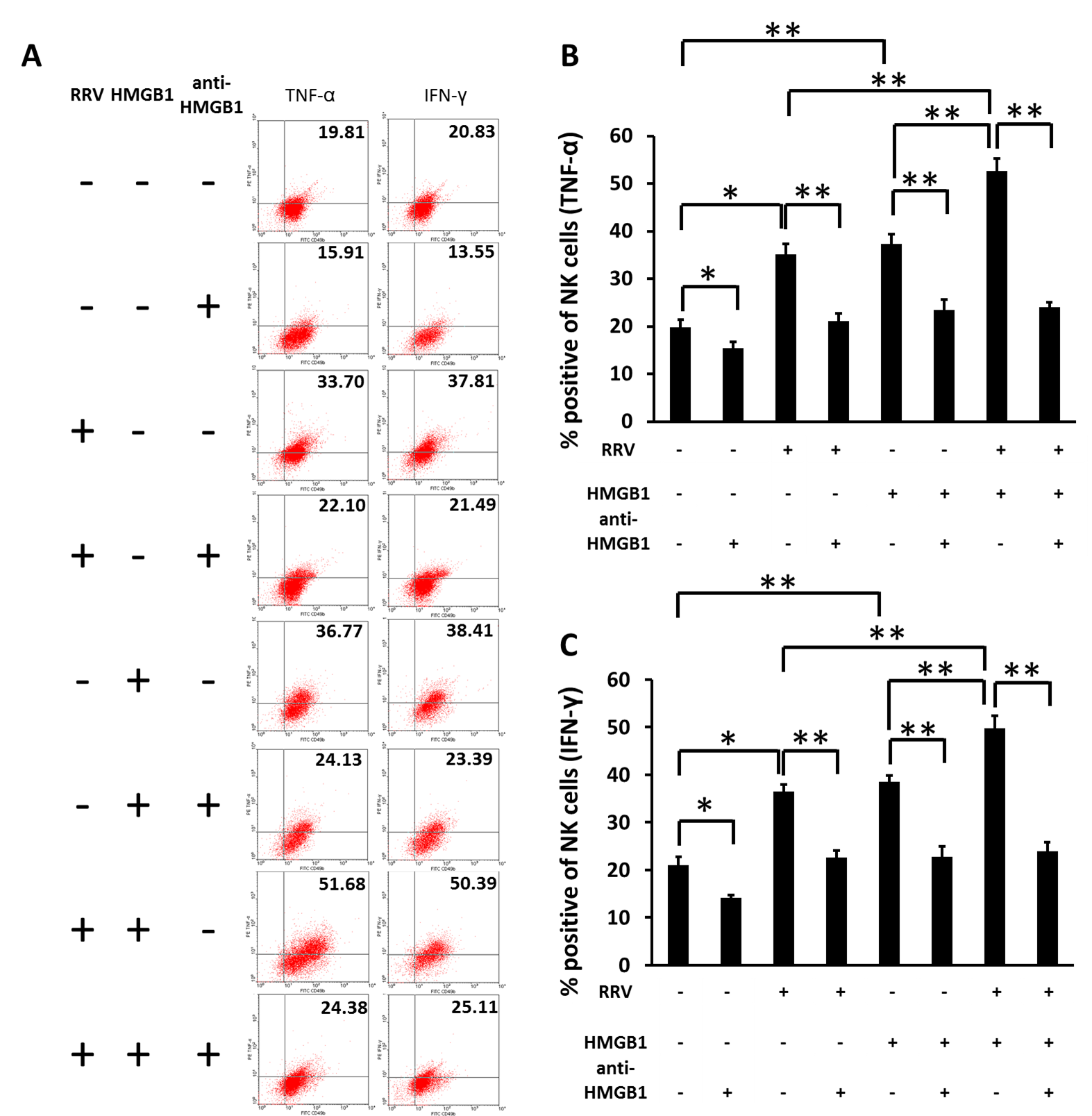

Supplement: Figure S2 — HMGB1 released from RRV-infected cholangiocytes promotes NK cell activation. (A) Flow cytometric analyses of expression of TNF-α and IFN-γ on CD49b+ NK cells stimulated by HMGB1. All NK cells were harvested from livers of adult wild-type B6 mice. The NK cells were treated with −/+ anti-HMGB1 antibody prior to −/+ HMGB1 stimulation, and the cholangiocytes were pretreated with −/+ RRV. Values in the right-upper quadrant of dot plots represent percent cells positive for CD49b and expression of TNF-α and IFN-γ of NK cells. The data are shown as representative dot plots. The average percentages of TNF-α+ and IFN-γ+ CD49b+NK cells are shown in B and C. (TIF) [file ppat.1004011.s002.tif]

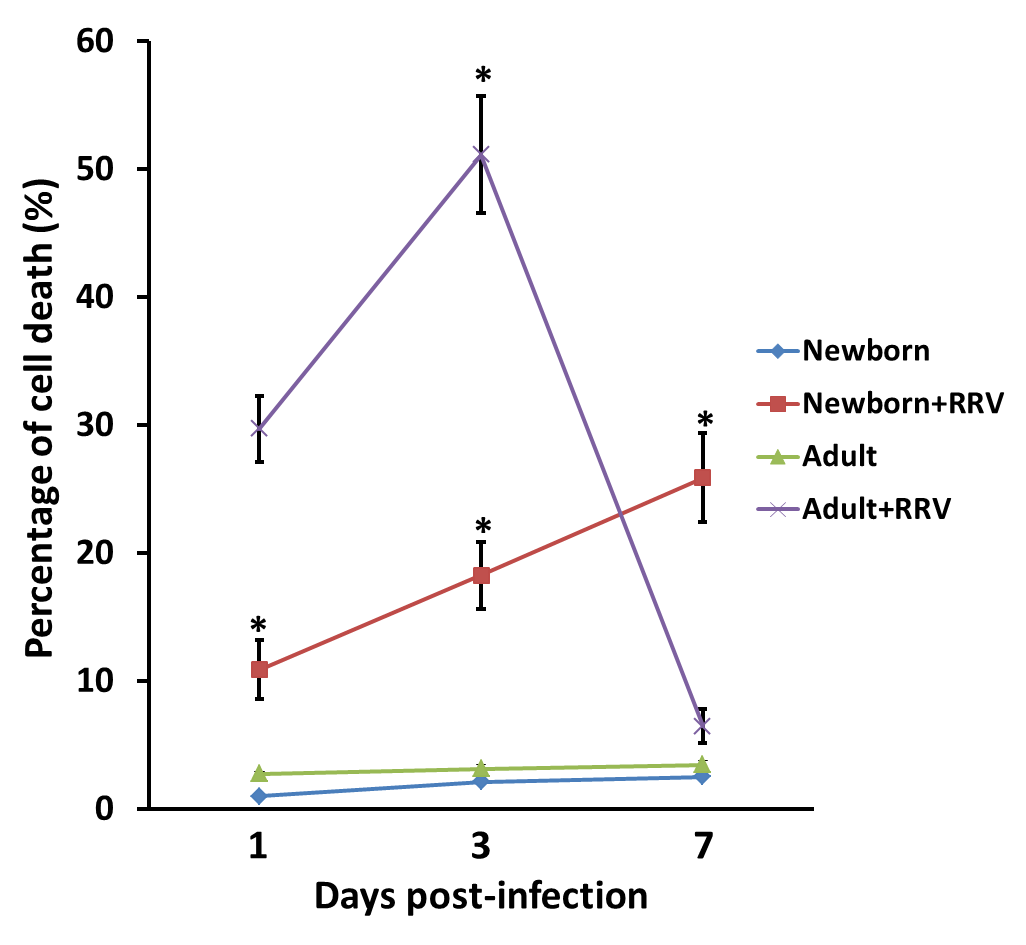

Supplement: Figure S3 — In vivo cytotoxicity assay of NK cells of RRV-infected mice at different ages. Cytotoxicity is measured by percentage of cholangiocyte death. NK cells were derived from newborn mice −/+ RRV infection or adult mice −/+ RRV infection. One day, 3 days and 7 days after RRV infection, livers of mice were used as the source of NK cells. N = 5 mice per group. The values represent the percentages of cholangiocyte death and are expressed as mean ± SD. (TIF) [file ppat.1004011.s003.tif]

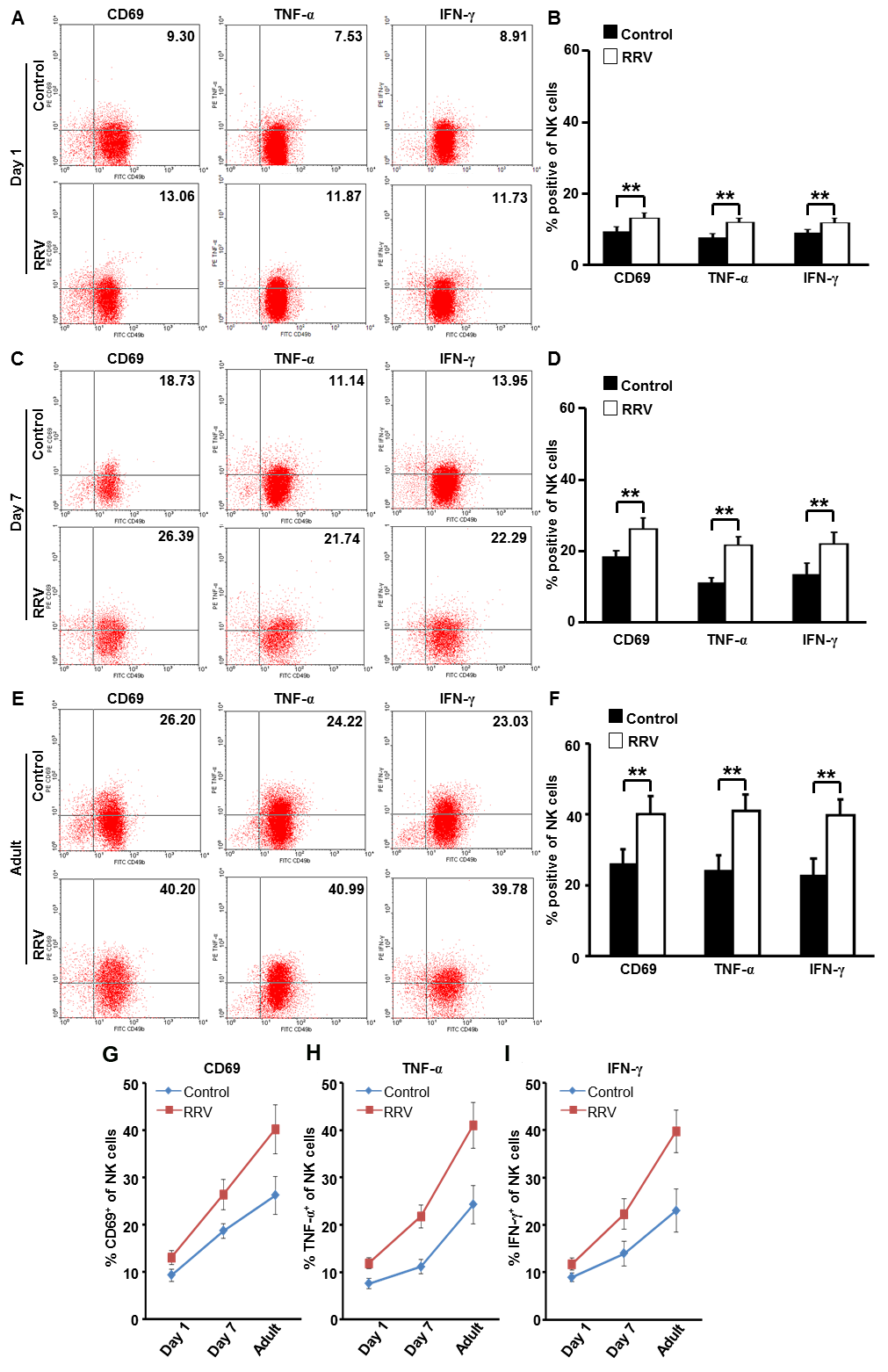

Supplement: Figure S4 — Age affects the RRV-induced activation of NK cells in vivo. (A, C and E) Flow cytometric analyses of activation markers of CD69, TNF-α and IFN-γ on CD49b+ NK cells in B6 mice challenged with RRV at different age groups (1 day old, 7 day old and 10 week old). Mice were injected with vehicle or RRV. NK cells were harvested from the livers of mice at 24 hours after RRV challenge. Values in the right-upper quadrant represent of dot plots percent cells positive for CD49b and activation markers of NK cells and the data are shown as representative dot plots. The average percentages of activation marker positive NK cells are shown in B, D and F. The change of percentages of CD69+ (G), TNF-α+ (H) and IFN-γ+ (I) NK cells and average percentage of activation marker positive NK cells in the 1 day, 7 day and adult groups was illustrated in line charts. **p<0.01; N = 5 mice per group. The values are expressed as mean ± SD. (TIF) [file ppat.1004011.s004.tif]

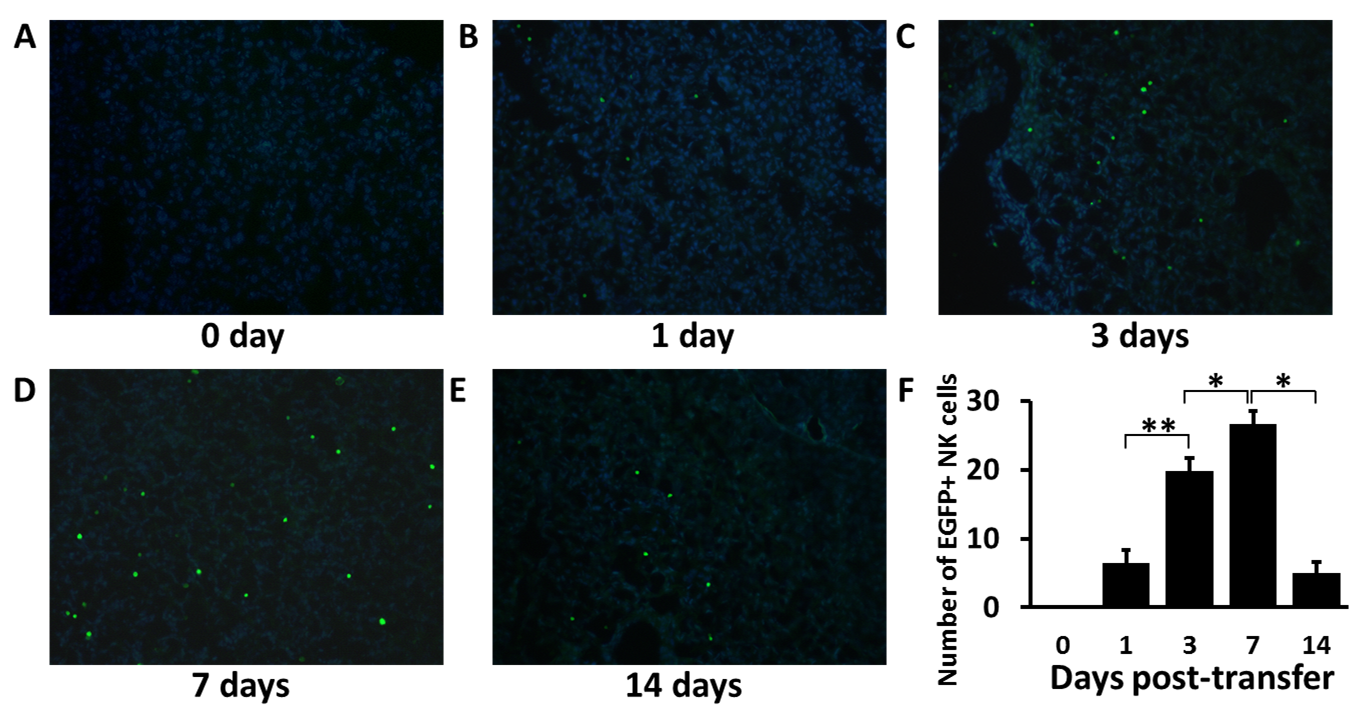

Supplement: Figure S5 — Tracking of transferred NK cells in the liver. The transferred EGFP NK cells were tracked in the liver at 0, 1, 3, 7 and 14 days post-transfer (A to E). Green fluorescence represents EGFP NK cells and the DAPI is used for counterstaining of nuclei. N = 5 mice per group. The magnification is 200×. (F) The values represent the average of EGFP NK cells in the liver per vision field and are expressed as mean ± SD. (TIF) [file ppat.1004011.s005.tif]

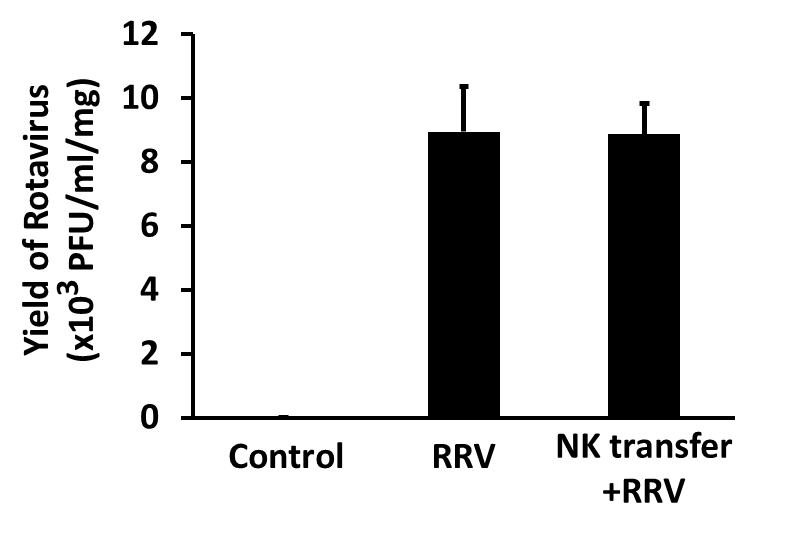

Supplement: Figure S6 — Plaque forming assay for detection of RRV in bile ducts. Viral titer in the control mice, RRV infected mice and RRV infected mice with prior NK cell transfer. The mean viral titer in the bile duct was expressed as mean±SD (103 PFU/ml/mg). (TIF) [file ppat.1004011.s006.tif]

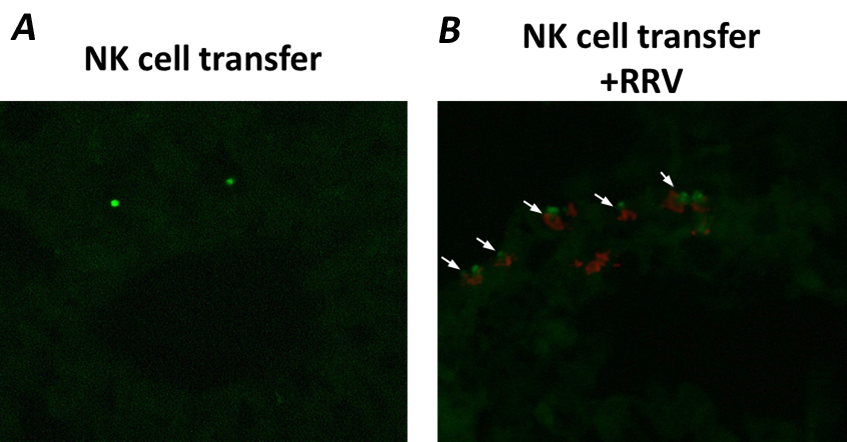

Supplement: Figure S7 — NK cells attaching to RRV infected cells. The transferred EGFP NK cells (green fluorescence) were tracked in the liver 24 hours after NK cell transfer in non-infected mice (A) or RRV-infected mice (B). VP4 staining (red fluorescence) represents for RRV infected cells in the biliary tree. The white arrows indicate the sites where the NK cells attach the RRV infected cells. (TIF) [file ppat.1004011.s007.tif]

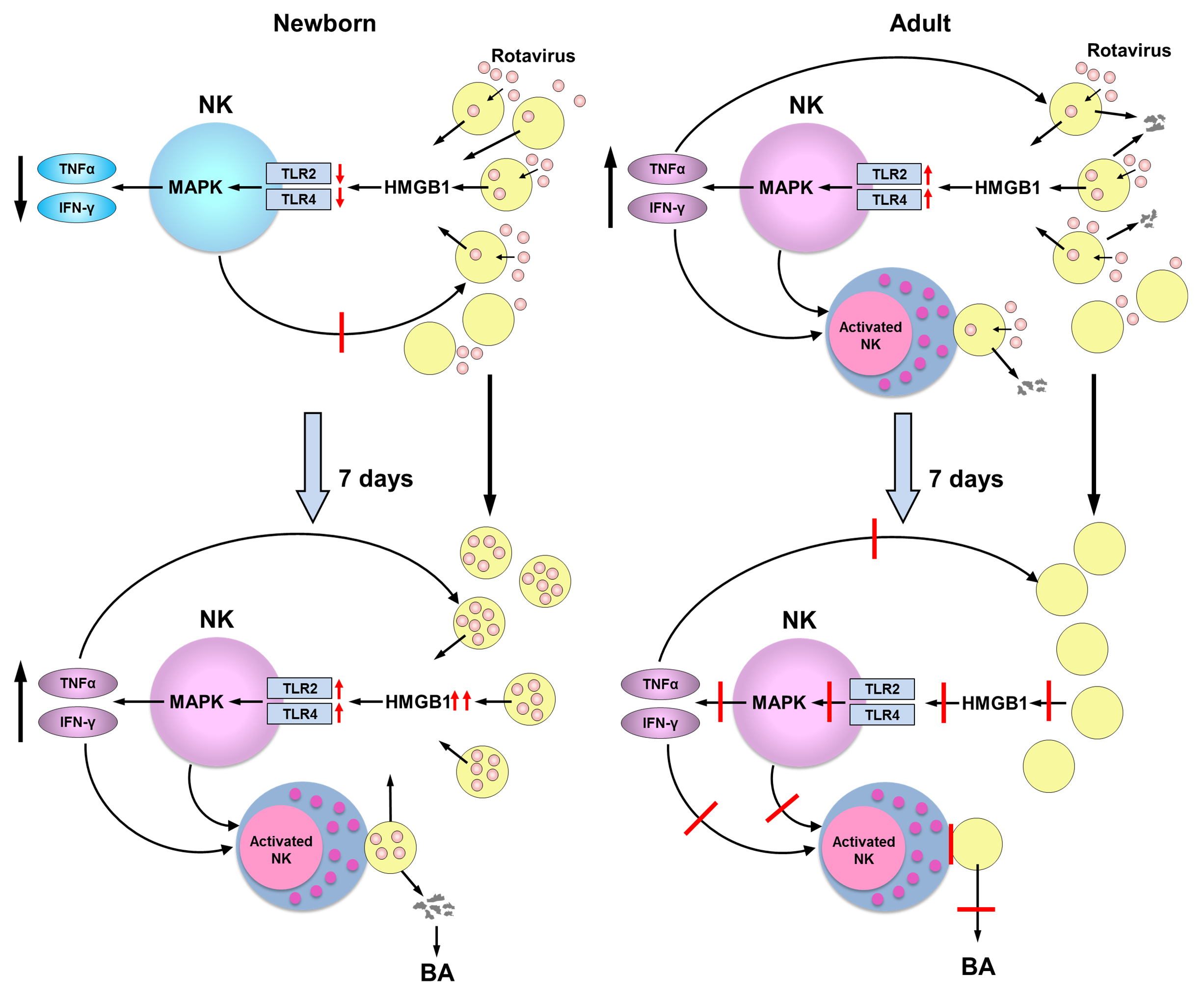

Supplement: Figure S8 — The relationship between maturation of NK cells and development of BA. After RRV infects bile ducts of 1 day old newborn mice, the cholangiocytes begin to secrete HMGB1. However, due to the low expressions of TLR2 and TLR4 on newborn NK cells, HMGB1 is incapable of activating NK cells via TLRs-MAPK signaling pathway. Thus, neither enough pro-inflammatory cytokines are secreted, nor is there enough NK cell cytotoxicity to eliminate RRV injured cholangiocytes. These factors cause persistent RRV infection in cholangiocytes of newborn mice. As mice age, they gain increased expressions of TLR2 and TLR4 on their NK cells and the persistently infected cholangiocytes persistently release HMGB1. These matured NK cells are capable of recognizing the increased HMGB1 signal, leading to activation of NK cells via the TLRs-MAPK signaling pathway. Activated NK cells secrete large amounts of pro-inflammatory cytokines, and generate persistent cytotoxicity on RRV-infected cholangiocytes, thus the cholangiocytes are persistently damaged by pro-inflammatory cytokines and direct NK cell cytotoxicity. With destruction of extrahepatic bile duct lumens, mice challenged with RRV at their neonatal stage consequently develop BA. On the other hand, the activation of NK cells of adult mice is increased, thus their mature NK cells eliminate RRV-infected cholangiocytes shortly after infection, which prevents any possibility of persistent RRV infection in bile ducts and further release of HMGB1. Therefore, neither the biliary tracts are further damaged nor does BA develop. (TIF) [file ppat.1004011.s008.tif]
