# Supplementary material for: HMGB1-Promoted and TLR2/4-Dependent NK Cell Maturation and Activation Take Part in Rotavirus-Induced Murine Biliary Atresia
Source: PLoS Pathog. 2014 Mar 20;10(3):e1004011. doi: 10.1371/journal.ppat.1004011 (PMC3961347; doi:10.1371/journal.ppat.1004011)
Supplement: Table S1 — Clinical data of patients diagnosed with biliary atresia (BA) and congenital dilation of the bile duct (CDB). (DOCX) [file ppat.1004011.s009.docx]

**Table S1** Clinical data of patients diagnosed with biliary atresia (BA) and congenital dilation of the bile duct (CDB)

| Disease | Cases | Gender | | Age at admission (days) | Age at operation (days) | Pre-operative total bilirubin (μmol/L) | Post-operative total bilirubin (μmol/L) | Pre-operative direct bilirubin (μmol/L) | Post-operative direct bilirubin (μmol/L) |
| --- | --- | --- | --- | --- | --- | --- | --- | --- | --- |
|  |  | Male | Female |  |  |  |  |  |  |
| BA | 9 | 5 | 4 | 53.4±20.7 | 80.9±11.9 | 158.0±33.8 | 106.3±20.8 | 124.4±30.4 | 82.4±16.8 |
| CDB | 5 | 2 | 3 | 89.2±32.3 | 101.2±38.4 | 13.3±6.1 | 7.1±2.2 | 7.5±3.1 | 4.3±1.6 |
