# Supplementary material for: HMGB1-Promoted and TLR2/4-Dependent NK Cell Maturation and Activation Take Part in Rotavirus-Induced Murine Biliary Atresia
Source: PLoS Pathog. 2014 Mar 20;10(3):e1004011. doi: 10.1371/journal.ppat.1004011 (PMC3961347; doi:10.1371/journal.ppat.1004011)
Supplement: Table S2 — Gene name and nucleotide composition for primers used in real-time RT PCR. (DOCX) [file ppat.1004011.s010.docx]

**Table S2** Gene name and nucleotide composition for primers used in real-time RT PCR

| **Gene** | **Species** | **Primer Sequences** | **Accession Number** |
| --- | --- | --- | --- |
| *HMGB-1* | Human | For: 5’- GCAAGCGAACAGCAGGGTTA-3’ | [NM_002128](http://www.ncbi.nlm.nih.gov/entrez/query.fcgi?cmd=Search&db=Nucleotide&term=NM_002128) |
|  |  | Rev: 5’-TCAGATTGAGTCATTTGCTCCTCTT-3’ |  |
| *TLR-2* | Human | For: 5’- GAAAGCTCCCAGCAGGAACATC-3’ | [NM_003264](http://www.ncbi.nlm.nih.gov/entrez/query.fcgi?cmd=Search&db=Nucleotide&term=NM_003264) |
|  |  | Rev: 5’-GAATGAAGTCCCGCTTATGAAGACA-3’ |  |
| *TLR-4* | Human | For: 5’- AGGATGATGCCAGGATGATGTC-3’ | NM_014817 |
|  |  | Rev: 5’-TCAGGTCCAGGTTCTTGGTTGAG-3’ |  |
| *Hmgb1* | Mouse | For: 5’- CGTCTGGCTCCCGCTCTCACA-3’ | [NM_010439](http://www.ncbi.nlm.nih.gov/entrez/query.fcgi?cmd=Search&db=Nucleotide&term=NM_010439) |
|  |  | Rev: 5’-GAGTCGCCCAGTGCCCGTC-3’ |  |
| *Tlr-2* | Mouse | For: 5’- GGGGTGTGTGATGGCCGCTC-3’ | [NM_011905](http://www.ncbi.nlm.nih.gov/entrez/query.fcgi?cmd=Search&db=Nucleotide&term=NM_011905) |
|  |  | Rev: 5’-TGGAGGTTCGCACACGCTCG-3’ |  |
| *Tlr-4* | Mouse | For: 5’- TGCTACAGCTCACCTGGGGCT-3’ | NM_021297 |
|  |  | Rev: 5’-TCTGCCCGGTAAGGTCCATGC-3’ |  |
| *VP4* | Rhesus- Rotavirus | For: 5’- GAAGCGGGAACAGATGGAAGA-3’ | AY033150 |
|  |  | Rev: 5’-TGGCTGAGATGACCGGAGAGT-3’ |  |
| *Egfp* | Mouse | For: 5’-GACAAGCAGAAGAACGGCATCAAG-3’ | U57607 |
|  |  | Rev: 5’-GCTTCTCGTTGGGGTCTTTGCT-3’ |  |
| *β-actin* | Mouse | For: 5’-CTGAGAGGGAAATCGTGCGT-3’ | NM_031144 |
|  |  | For: 5’-CCACAGGATTCCATACCCAAGA-3’ |  |
| *GAPDH* | Human | For: 5’-TGCACCACCAACTGCTTAGC-3’ | [M19533](http://www.iovs.org/external-ref?link_type=GEN&access_num=M19533) |
|  |  | Rev: 5’-GGCATGGACTGTGGTCATGAG-3’ |  |
