# Supplementary material for: HMGB1-Promoted and TLR2/4-Dependent NK Cell Maturation and Activation Take Part in Rotavirus-Induced Murine Biliary Atresia
Source: PLoS Pathog. 2014 Mar 20;10(3):e1004011. doi: 10.1371/journal.ppat.1004011 (PMC3961347; doi:10.1371/journal.ppat.1004011)
Supplement: Table S3 — Antibodies for immunohistochemical or immunofluorescent staining. (DOCX) [file ppat.1004011.s011.docx]

**Table S3** Antibodies for immunohistochemical or immunofluorescent staining

| **Antibody** | **Host species** | **React with** | **Catlog number** | | **Company** |
| --- | --- | --- | --- | --- | --- |
| HMGB-1 | Rabbit | Human/Mouse | Ab18256 | Abcam, Cambridge, Massachusetts, USA | |
| TLR-2 | Rabbit | Human/Mouse | T0337 | Epitomics, Burlingame, California, USA | |
| TLR-4 | Rabbit | Human/Mouse | T0342 | Epitomics, Burlingame, California, USA | |
