# Supplementary material for: HMGB1-Promoted and TLR2/4-Dependent NK Cell Maturation and Activation Take Part in Rotavirus-Induced Murine Biliary Atresia
Source: PLoS Pathog. 2014 Mar 20;10(3):e1004011. doi: 10.1371/journal.ppat.1004011 (PMC3961347; doi:10.1371/journal.ppat.1004011)
Supplement: Table S4 — Antibodies for western blotting. (DOCX) [file ppat.1004011.s012.docx]

**Table S4** Antibodies for western blotting

| **Antibody** | **Host species** | **React with** | **Molecular Weight** | **Catlog number** | **Company** |
| --- | --- | --- | --- | --- | --- |
| HMGB-1 | Rabbit | Human/Mouse | 29 kDa | Ab 18256 | Abcam, Cambridge, Massachusetts, USA |
| TLR-2 | Rabbit | Human/Mouse | 89 kDa | T0337 | Epitomics, Burlingame, California, USA |
| TLR-4 | Rabbit | Human/Mouse | 96 kDa | T0342 | Epitomics, Burlingame, California, USA |
| p38 | Rabbit | Mouse | 43 kDa | 21245 | Signalway Antibody, Pearland, Texas, USA |
| p-p38 | Rabbit | Mouse | 43 kDa | 11253 | Signalway Antibody, Pearland, Texas, USA |
| ERK | Rabbit | Mouse | 42/44 kDa | 21237 | Signalway Antibody, Pearland, Texas, USA |
| p-ERK | Rabbit | Mouse | 42/44 kDa | 11245 | Signalway Antibody, Pearland, Texas, USA |
| JNK | Rabbit | Mouse | 46/54 kDa | 21241 | Signalway Antibody, Pearland, Texas, USA |
| p-JNK | Rabbit | Mouse | 46/54 kDa | 11249 | Signalway Antibody, Pearland, Texas, USA |
| β-actin | Mouse | Human | 42 kDa | A2228 | Sigma-Aldrich Corp, St Louis, Missouri, USA |
| β-Tublin | Mouse | Mouse | 55 kDa | T4026 | Sigma-Aldrich Corp, St Louis, Missouri, USA |
